# Application of phage display to high throughput antibody generation and characterization

**DOI:** 10.1186/gb-2007-8-11-r254

**Published:** 2007-11-29

**Authors:** Darren J Schofield, Anthony R Pope, Veronica Clementel, Jenny Buckell, Susan DJ Chapple, Kay F Clarke, Jennie S Conquer, Anna M Crofts, Sandra RE Crowther, Michael R Dyson, Gillian Flack, Gareth J Griffin, Yvette Hooks, William J Howat, Anja Kolb-Kokocinski, Susan Kunze, Cecile D Martin, Gareth L Maslen, Joanne N Mitchell, Maureen O'Sullivan, Rajika L Perera, Wendy Roake, S Paul Shadbolt, Karen J Vincent, Anthony Warford, Wendy E Wilson, Jane Xie, Joyce L Young, John McCafferty

**Affiliations:** 1 Abcam Ltd, Cambridge Science Park, Cambridge CB4 0FW, UK; 2 Wellcome Trust Sanger Institute, Genome Campus, Hinxton, Cambridgeshire CB10 1HH, UK; 3 Cambridge Antibody Technology, Granta Park, Cambridge CB21 6GH, UK; 4 Cancer Research UK, Research Monoclonal Antibody Services, Lincoln's Inn Fields, London WC2A 3PX, UK; 5 Molecular Products Ltd, Thaxted CM6 2LT, UK; 6 Department of Biochemistry, University of Cambridge, Downing Site, Cambridge CB2 1QW, UK; 7 Cellular Histopathology Department, Bedford Hospital NHS Trust, Bedford MK42 9DJ, UK; 8 Cancer Research UK, Cambridge Research Institute, Cambridge CB2 0RE, UK; 9 GlaxoSmithKline Medicines Research Center, Stevenage SG1 2NY, UK; 10 Genzyme Therapeutics Ltd, Cambridge Science Park, Cambridge CB4 0WG, UK; 11 Novartis Institutes for BioMedical Research, Discovery Technologies, CH-4002 Basel, Switzerland; 12 Astra Zeneca Innovation Centre China, Shanghai, 200041, China

## Abstract

We have created a high quality phage display library containing over 10^10 ^human antibodies and describe its use in the generation of antibodies on an unprecedented scale. We have selected, screened and sequenced over 38,000 recombinant antibodies to 292 antigens, yielding over 7,200 unique clones. 4,400 antibodies were characterized by specificity testing and detailed sequence analysis and the data/clones are available online. Sensitive detection was demonstrated in a bead based flow cytometry assay. Furthermore, positive staining by immunohistochemistry on tissue microarrays was found for 37% (143/381) of antibodies. Thus, we have demonstrated the potential of and illuminated the issues associated with genome-wide monoclonal antibody generation.

## Abstract 2

A phage display library has been constructed containing over 1010 human antibodies, allowing the large-scale generation of antibodies. Over 38,000 recombinant antibodies against 292 antigens were selected, screened and sequenced, and 4,400 resultant unique clones characterized further.

## Background

The availability of multiple genome sequences provides a valuable reference facilitating systematic family-wide or even genome-wide investigation of gene function. Information on gene structure, evolution and family relationships can be drawn and predictions of biochemical function can be made through sequence comparisons. Functional processes in cells, however, are driven by proteins and a deeper understanding of gene function will ultimately require information on protein interactions, protein expression levels, modifications and sites of action. Antibodies provide a valuable means of gaining such information. Several initiatives to generate monoclonal antibodies on a genome-wide scale are under consideration [1,2]. Large scale profiling of commercial and newly generated polyclonal antibodies to over 700 antigens has previously been described [3]. Panels of monoclonal antibodies, however, would have advantages over polyclonal antibodies by being a renewable resource of defined, homogeneous composition. Potential cross-reactivity will be less than in a complex polyclonal mixture. Furthermore, the availability of multiple independent antibodies, as shown here, allows independent verification of results.

Generation of antibodies on such a scale presents a range of challenges, spanning the generation of antigen through generation and validation of antibodies to production, tracking and application in a relevant biological read-out. One of the first bottlenecks is the creation of quality recombinant protein in high throughput. This goal requires methods for primer design, cloning, sequence confirmation, protein expression, purification and quality control of the resulting products. In this study, protein products derived from both bacterial and mammalian systems were used as targets for antibody generation. *Escherichia coli *provides an efficient system for protein expression, and generation of soluble product can be aided by addition of solubility enhancing and affinity purification tags [4]. In addition, a protein expression system based on transient transfection of mammalian cells [5] was used for expression of receptor extracellular domains [6].

Phage display is a scalable method of generating antibody reagents, and phage-antibody libraries can provide a rich source of antibody diversity, potentially providing hundreds of unique antibodies per target. The antibody gene, once isolated, can be conveniently shuttled into a variety of expression formats, providing a renewable resource of antibody protein [7]. We report here the generation of an antibody phage display library of over 10^10 ^clones and its application to the selection and screening of over 38,000 antibody clones. DNA sequencing allows redundancy to be removed from the antibody panel and permits a definitive description of the resulting antibody gene and its product. Over 7,200 unique recombinant antibodies to 290 targets were identified. Of these, 4,437 were picked and their specificity determined against a wider panel of antigens. In addition, detection sensitivity was measured for 100 antibodies to 10 antigens using a bead based flow cytometry assay, with sensitivity below 18,000 antigens/bead demonstrated for all 10 antigens. This assay was also predictive of performance in detecting endogenous levels of antigen by flow cytometry. Finally, we illustrate their application in immunohistochemistry using tissue microarrays to produce protein expression profiles.

Thus, we demonstrate the potential of high throughput processes for the generation and validation of recombinant proteins and antibodies. We illustrate examples of information, such as cross-reactivity, sequence, and performance data, that may form part of a simple standardized validation protocol. Apart from exemplifying the potential of such large scale approaches, the validated antibody and protein reagents generated in this study will have research and diagnostic potential and have been made available, along with the characterization data, to the scientific community [8,9].

## Results

### High-throughput antibody selection

We report the construction of an antibody phage display library of 1.1 × 10^10 ^clones and its utilization for high throughput antibody generation and characterization. The antibody library was created by sequentially cloning a repertoire of light chain variable regions (VL) followed by cloning of heavy chain variable regions (VH). The heavy and light chain repertoires were created by PCR amplification from human lymphocytes from 43 donors, mainly collected from peripheral blood. Human VH genes are grouped into seven families and VL genes into six kappa and ten lambda families, based on sequence homologies. PCR primers were designed with reference to these VH and VL germline families [10]. The full set of primers along with detailed protocols for their use are listed in Additional data file 1.

**Figure 1:**
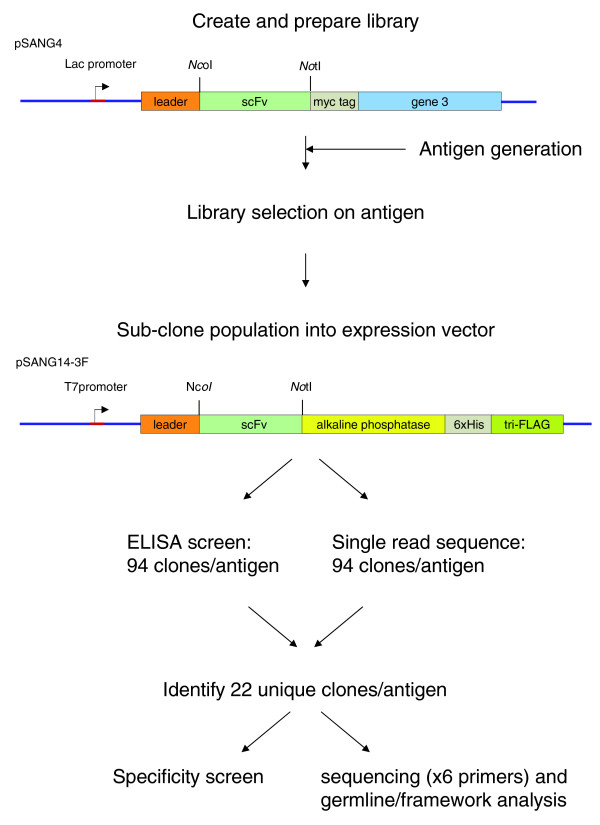
Schematic representation of the process for the high throughput generation and screening of recombinant antibodies.

In a first step, the VL repertoire was cloned into the *Nhe*I/*Not*I sites of pSANG4 (Figure 1). Subsequently, plasmid DNA was prepared from this library before cloning the VH repertoire into the *Nco*I/*Xho*I sites. The final format of the antibody is a single chain Fv (scFv) with VH and VL fragments joined by a flexible linker peptide (Gly_4 _Ser Gly_4 _Ser Gly_3 _Ala Ser).

The library was generated using B lymphocytes from 42 human peripheral blood donations and 1 tonsil. Diversity was maintained at a maximum by a variety of strategies. All donations were maintained separately and combined to 11 pools at the mRNA stage. V region primers (14 heavy chain, 13 kappa light chain and 15 lambda light chains) were kept separate during primary PCR for each of the 11 mRNA pools, that is, 462 separate primary PCR reactions were performed. Each of the kappa light chain families and three pools of the lambda chain families were transformed separately and only pooled into two master sets (kappa and lambda) once DNA was isolated from each library stock to prepare vector for heavy chain cloning. Transformation of each of the 7 heavy chain families into both the kappa and lambda light chain libraries was done separately creating a total of 14 pools of scFv libraries, supplemented with 2 additional sub-libraries representing VH3/Vkappa1 and VH/kappa3 combinations. Finally, phage were prepared individually from each of the 16 different aliquots and pooled to maintain equivalent representation of each sub-aliquot.

Phage particles were rescued using a trypsin sensitive helper virus [11]. Thus, phage particles incorporating the minor coat protein encoded solely from the helper phage are susceptible to trypsin cleavage and will not be infective. In contrast, those displaying an antibody-gene 3 fusion will retain infectivity after trypsin treatment. This reduces background from 'non-participating' phage particles that have been generated without utilizing an antibody-gene 3 product from the library. This increased the efficiency of binder isolation such that we routinely required only two rounds of selection. High throughput selection was further facilitated using a liquid culture amplification/rescue method, eliminating the need for plating out and scraping of bacterial culture plates.

Antibodies were selected to 404 antigen targets (representing 280 genes) that were primarily produced in *E. coli *[4] or in mammalian cells [6], with a proportion sourced commercially. The majority of genes (214/280) were cell surface receptors, with 30% of the immunoglobulin superfamily represented (139 genes). Receptors were chosen as they comprise a major class of drug targets that provide information on the communication potential of cells, and act as identifiers of different cell types, which can be used in their purification. Most of the genes were murine in origin (81%), with the remainder being human (18%) and rat (1%). A full gene list is provided at the 'Antibody Atlas db' [8] and in Additional data file 2.

Following selection, resultant antibody populations were sub-cloned into an expression vector (pSANG14-3F), which fuses the antibody scFv to a tri-FLAG tag sequence and the dimeric enzyme bacterial alkaline phosphatase. This provides a convenient enzymatic tag to detect binding as well as driving dimerization, thereby increasing avidity of binding [7]. From each selection, 94 clones were screened and sequenced, and a sub-set of up to 22 unique clones identified for further specificity and sequence characterization. The process is outlined in Figure 1.

### Primary screening and sequencing of selected antibodies

The primary screening assay to identify binders was initially performed with bacterial lysates in a 96-well format using direct detection of alkaline phosphatase fused to the antibody fragment. This convenient assay requires no secondary antibody and involves a single cycle of binding and washing of the antibody-alkaline phosphatase fusion, followed by addition of enzymatic substrate. In subsequent work, the assays were performed in a 384-well format using europium labeled secondary reagents, to more closely reflect the system we used for secondary screening (see below).

To identify unique binders, all primary clones were sequenced using a single primer that annealed upstream of the 5' end of the VH gene. In total, 38,164 clones were screened and sequenced. Of these, 9,384 were positive in primary screening ELISA. Sequence analysis of all of these identified 7,236 unique clones. With 292 antigens passing selection, this represents a success rate of 72% with an average of 25 clones for each positive antigen.

**Figure 2:**
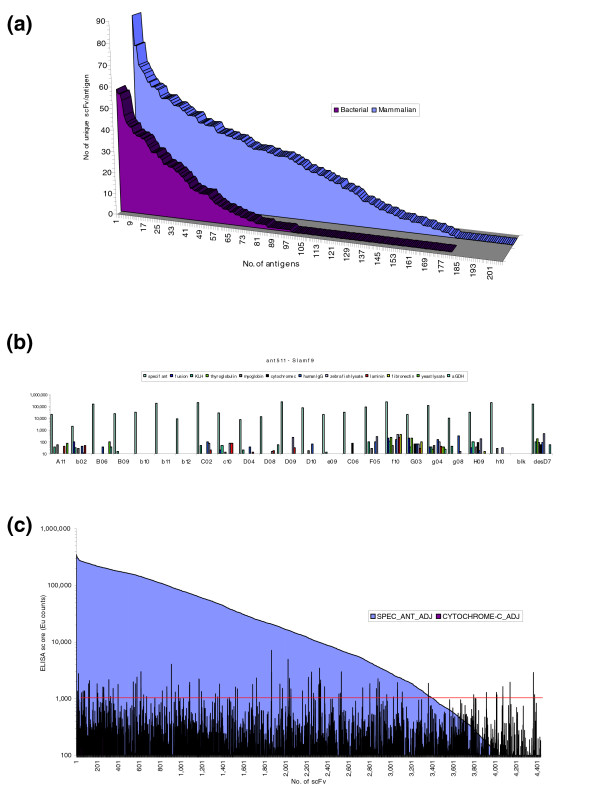
High throughput generation and characterization of recombinant antibodies. **(a) **For each target antigen, 94 clones were screened in ELISA and sequenced to identify unique clones. The plot shows the number of antigens selected (x-axis) versus the number of unique positive antibodies generated (y-axis) for each antigen. Separate plots are presented for antigens produced in either bacterial or mammalian expression systems, illustrating the improved success rate for mammalian antigens. **(b) **Example specificity data for antibodies selected against Slam f9 (produced in the HEK293 mammalian expression system). All antibodies are screened against target antigen, the relevant fusion partner that was used in selection, keyhole limpet hemocyanin (KLH), thyroglobulin, myoglobin, cytochrome c, human IgG, laminin, fibronectin, α-glycerol phosphate dehydrogenase, and total protein lysates from zebra fish (*D. rerio*) and yeast (*S. pombe*). Results are shown for 22 different antibodies as well as our routine anti-desmin control (des-D7) and a no antibody control. Detection was via time resolved fluorescence and values are shown on a logarithmic scale. **(c) **Global summary of secondary ELISA data for all antibodies in secondary screening. Signal generated on specific antigen is shown for all 4,437 samples (solid block). Signal achieved on one of the ten irrelevant antigens (cytochrome c) is also shown.

Figure 2a shows the number of unique scFv binders generated for each target, demonstrating the diversity achieved from the library. This figure compares the success rates of protein generated in both bacterial and mammalian expression systems. It is clear that the performance of targets derived from mammalian expression systems were superior in terms of generating target specific, sequence unique scFvs. For bacterially expressed antigens, 54% of the targets gave rise to one or more unique binders after the primary screening ELISA stage, with 19% giving rise to 22 or more unique clones. For mammalian expressed antigens, 82% of the targets gave rise to one or more unique binders after the primary screening ELISA, with 52% giving rise to 22 or more unique clones. The diversity of antibodies generated from the library across a wide range of antigens clearly illustrates the quality of the library and methods used. Furthermore, it suggests that more screening would generate a greater diversity of antibodies. Generation of hundreds of antibodies to a single antigen using a similar antibody phage display library has previously been described [12].

To identify panels of antibodies for further study, sequences were clustered according to the heavy chain complementarity determining regions (CDRs) with a particular focus on CDR3, which is most variable. Clones were also ranked according to signal-to-noise ratio in primary screening ELISA with priority given to clones with the highest signal-to-noise ratio and most divergent HCDR3 amino acid sequences. Up to 22 scFv clones were chosen for each target, yielding 4,437 antibodies to 286 antigens for specificity testing and high quality sequencing.

**Figure 3:**
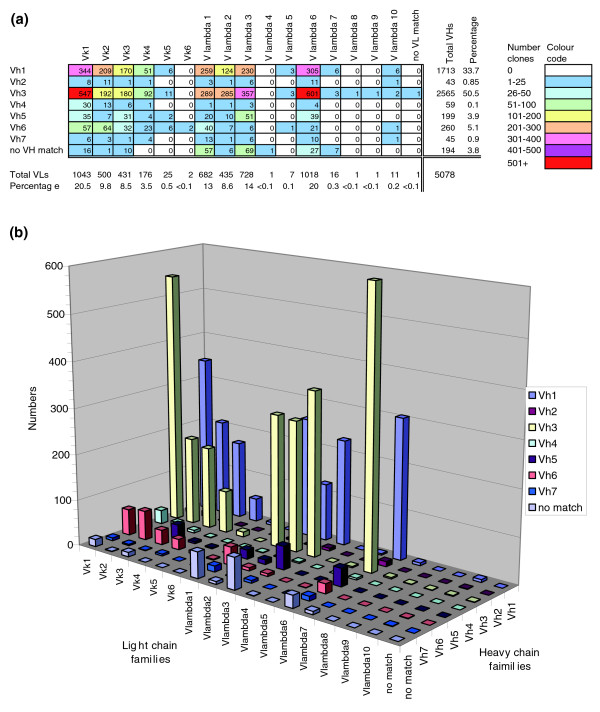
Frequency of VH and V kappa/V lambda germline gene combinations selected from the library. High quality sequence of all 4,437 clones undergoing secondary screening was generated by sequencing with six primers covering the VH and VL gene segments in both forward and reverse orientations. A consensus sequence was generated and the most closely related antibody germ line genes were identified. **(a) **Frequency of different combinations of VH and V kappa/V lambda germ-line genes occurring in the selected antibodies, represented both numerically and by color coding. **(b) **Frequency of different combinations of VH and V kappa/V lambda germline genes among the selected antibodies.

To give a definitive description of each antibody, all 4,437 clones undergoing secondary screening were sequenced in depth with 6 primers covering the VH and VL gene segments in both forward and reverse orientation. A consensus sequence was generated and the most closely related VH and VL germ line sequences were identified (Figure 3). This was done by comparing the VH and VL segment of each clone against a database of human antibody germline genes. The database uses 51 VH germline genes (across 7 sub-families, VH1-VH7), 40 germline V kappa genes (across the 6 sub-families Vkappa 1-Vkappa 6) and 31 germline V lambda genes (across the 10 V lambda families) [10]. Analysis shows that all VH, Vkappa and V lambda sub-families were represented in the selected population and most individual germline genes were identified as the closest hit in at least one selected clone. Thus, the full repertoire of antibody germline genes is being accessed during selection of the library. Figure 3 shows the frequency of different combinations of the VH, Vkappa and V lambda sub-families in the selected repertoire. Although the relative contribution of each germline family was normalized in the construction of the library, the results show a disproportionate occurrence of certain germline families (VH1, VH3, Vkappa 1, Vlambda 6). Consistent with previous work, this may reflect a greater degree of antibody diversity within these families in the initial library or may reflect emergence of clones during selection or screening as a result of improved expression from these families [13]. As well as assigning the closest germline gene for each clone, a detailed analysis was conducted to identify position and sequence of the framework and CDRs and this information is available at the Antibody Atlas db [8].

### Secondary specificity screening

The selected clones were tested in a secondary screening assay in order to confirm the results of the primary screening ELISA, and to identify antibody clones that were cross-reactive with a panel of irrelevant proteins. Each antibody clone was tested against the target antigen, the fusion partner used, eight irrelevant purified proteins and total protein lysates from zebrafish and yeast, representing more complex non-mammalian protein mixes. All secondary specificity screens took advantage of the sensitivity and dynamic range of time resolved fluorescence using europium labeled secondary antibodies. Figure 2b shows a representative cross-reactivity profile on all 12 antigens, for 22 antibodies generated to a single antigen. In a global view of the data, Figure 2c summarizes the range of ELISA signals achieved on secondary screening for all 4,437 clones on their specific antigen compared with the signal generated from one of the irrelevant antigens (cytochrome c). This illustrates that the signals achieved span three orders of magnitude. The signal level achieved is dependant on multiple factors, including efficiency of antigen coating, expression level of each antibody and the affinity of that antibody for antigen. Using 1,000 fluorescence units as a threshold, 76.6% of clones undergoing secondary screening were considered positive. Within this group, 80% of clones were specific for target antigen (defined as absence of signal above 1,000 units on all other antigens). Twelve percent of cross-reactivity assay failures at this stage were from antibodies that recognized the antigen fusion partners. Thus, although deselection with the fusion partner reduced the proportion of fusion binders generated, it did not completely prevent such antibodies arising.

The above analysis shows that 23% of clones deemed positive on primary screening were scored as negative on secondary screening. For many this was due to the increased stringency in the secondary screen since many of these failures were also the poorest performers in the primary screen. For five antigens, all antibody clones were negative on secondary screening, and in these cases the antigen was assumed to have precipitated over time in storage. This was substantiated in one case where the clones were positive on the same antigen in an alternative antigen construct (not shown).

Mirroring the primary screening results, antibodies to mammalian-produced antigens had a higher success rate in secondary screening, where 64% of antibodies generated were confirmed as antigen specific and, of those, 69% were scFv with high secondary ELISA scores (defined as 10-fold above the cut off, that is, 10,000 Europium units). In contrast, 57% of scFv generated to bacterially produced antigens were confirmed as antigen specific on secondary screening and, of those, 54% had high secondary ELISA scores. Analyzing the data by antigen target showed that 88% of all targets had at least one antigen-specific binder in the cross-reactivity assay, and 89% of these had at least one binder with high secondary ELISA score. In conclusion, secondary screening has confirmed the results of the initial high throughput screen for the majority of clones and has demonstrated high specificity for the target antigen in 80% of cases.

### Assessing performance of antibodies

**Figure 4:**
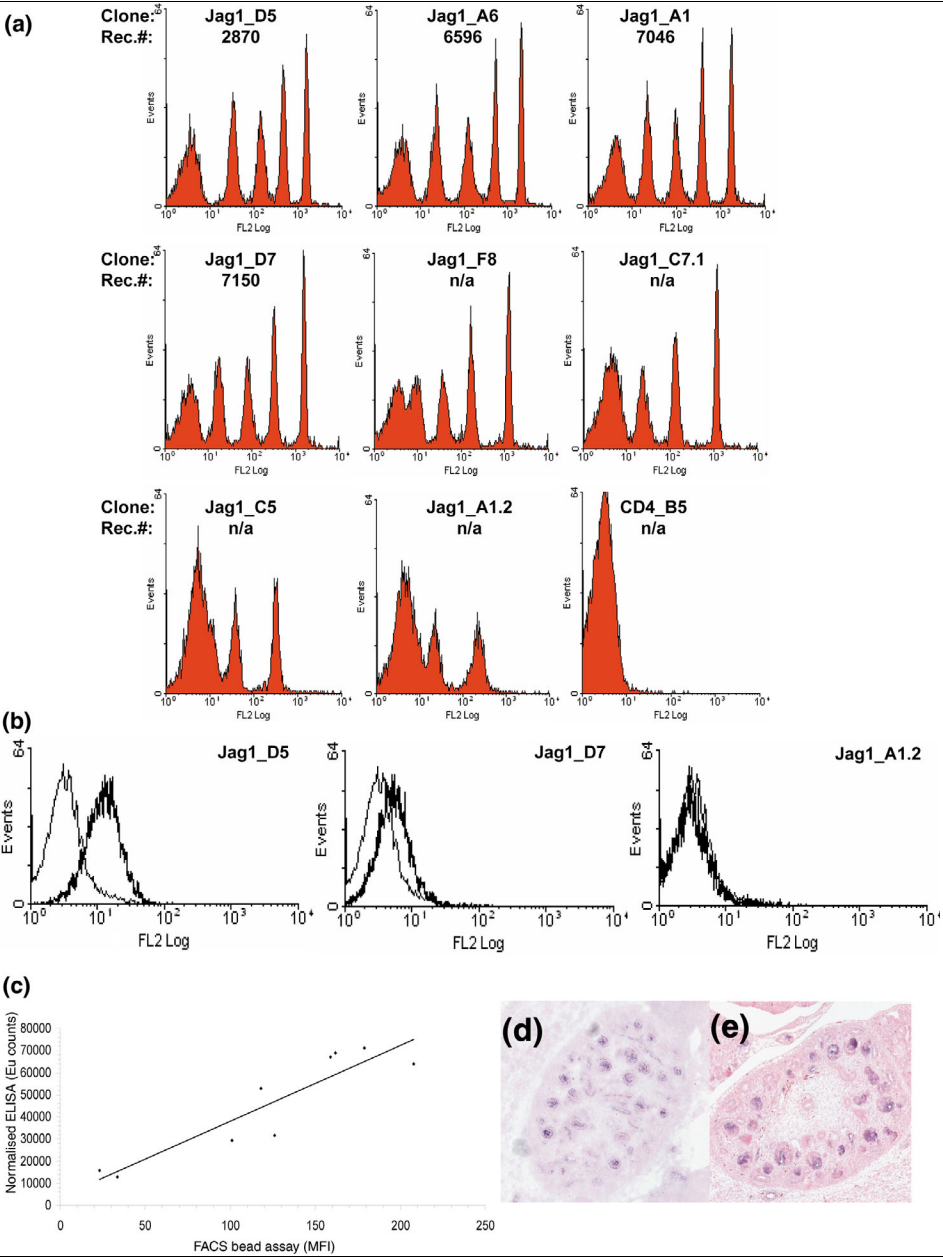
Assessing performance of a panel of anti-Jagged-1 antibodies. **(a) **Flow cytometry calibration beads with varying number of anti-human Fc antibodies were coated with Jagged-1-Fc fusion to yield antigen display levels of 29,000, 83,000, 204,000 and 619,000 Jagged-1 molecules/bead. These were labeled with a panel of different recombinant antibodies raised against Jagged-1 and binding was detected with labeled anti-FLAG antibodies. The resulting histograms are shown, giving different levels of sensitivity. In Jag1_D5 for example, five peaks are visible corresponding to uncoated beads and each of the four antigen coated beads. In the case of Jag1_C5, where there is lower sensitivity, only the two beads with highest density are resolved while the others merge with that of the uncoated bead. Where all four beads are clearly resolved, we have calculated the theoretical limit of detection of receptors per bead (Rec. #). **(b) **46C mouse embryonic stem cells were stained with the panel of Jagged-1 antibodies and analyzed by flow cytometry. Staining for three antibodies (Jag1_D5, Jag1_D7 and Jag1_A1.2) is shown. The sensitivity of each corresponds to that seen in the bead assay above. **(c) **A plot of normalized ELISA scores with performance in the bead-based flow cytometry performance assay for nine Jagged-1 specific antibodies. ELISA was carried out using 1 μg/ml of purified antibody. The mean time resolved fluorescence score (expressed as Eu counts) was plotted against median fluorescent intensity determined for an antigen density of 618,888 antigens/bead. The line represents a linear regression analysis of the data (R^2 ^value = 0.8323). **(d) **Immunohistochemical (Jag1_A6 scFv antibody) and **(e) ***in situ *hybridization staining of Jagged-1 in developing kidney of E14.5 mouse embryo, demonstrating localization of staining (dark purple) in developing renal tubules.

Although affinity is a useful definitive measurement in describing the properties of an antibody, measurement of absolute affinities for thousands of clones is impractical. A relative ranking of clones is possible using inhibition ELISA [14] or, more simply, with an end-point ELISA, provided the antibody concentration is normalized and the signal is within the dynamic range of the detection system. The ultimate measure of antibody utility, however, relates to performance and sensitivity in specific assays such as flow cytometry. When using biological samples, however, many factors apart from affinity affect the outcome, for example, level of antigen expression, modification of targets, effect of fixation, and so on. To assess the performance of a panel of antibodies in flow cytometry, independently of these factors, we have employed an assay using beads with defined antigen capture capacity ranging from 0 to >600,000 copies per bead. The read out from this assay gives an indication of the potential level of sensitivity a given antibody may have in cell based flow cytometry. Figure 4a shows staining of Jagged-1 coated beads, illustrating detection capability below 10,000 copies per bead for 4/9 antibodies. Normalized ELISA provides an alternative means of ranking antibodies and Figure 4c demonstrates a correlation between signal achieved in normalized ELISA and performance in the bead based assay.

Using Jagged-1 as an example, we show that performance in the bead assay in turn correlates with ability to detect endogenous levels of antigen. Jagged-1, which is expressed on embryonic stem cells [15] is a ligand for Notch receptors. The Notch signaling system is an ancient and widely used pathway in metazoan development and evolution, where it links the fate of one cell to its neighbors. Notch signaling involves a bi-molecular interaction between receptor and ligand on opposing cells. Mammalian notch signaling involves a choice of four notch receptors (Notch 1-4) and five ligands (Jagged 1,2, delta 1,3,4). Flow cytometry analysis of ES cells stained with three different anti-Jagged-1 antibodies is shown in Figure 4b. This shows that signal intensity varies with different antibodies and this, in turn, corresponds to the performance ranking found in the bead based assay.

**Figure 5:**
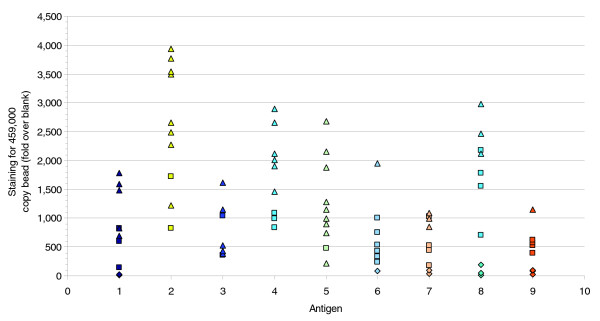
Detection sensitivity in a bead-based performance assay for a panel of antigens. The sensitivity limits of 90 antibodies to 9 different antigens were tested using a mix of antigen coated beads of different antigen densities. To summarize these data, the relative median fluorescent intensity on the bead comprising 459,000 antigen copies per bead was calculated (actual values and clone IDs given in Additional data file 2) and the ratio relative to uncoated beads plotted. To further illustrate sensitivity achieved, the identity of the lowest density bead that could be detected is indicated by the data label according to the following guide: 459,000 antigen copies per bead only (diamonds), down to 57,000 antigen copies per bead (square), and down to 18,000 antigen copies per bead (triangles). The antigens were: 1, Efna2; 2, Efna4; 3, Plaur; 4, Alcam; 5, Il13ra1; 6, Sigrr; 7, Ngfr; 8, Cd22; and 9, Vcam1.

The bead assay was applied to 90 other antibodies representing 9 different target genes. In this experiment beads were coated with antigen at a range of densities (18,000, 57,000, 459,000 antigens/bead). To help summarize and present the results for all 90 antibodies, the median fluorescent intensity for the bead coated with 459,000 target molecules was calculated. The signal relative to that of uncoated bead is plotted in Figure 5 and shows a wide range of signals up to 4,000-fold over background (values and antibody names are given in Additional data file 3). Detection of beads coated with 18,000 copies per bead was achieved with at least one antibody for all 9 genes tested. The sensitivity limit is illustrated in Figure 5 by using different data point symbols to indicate the lowest antigen density detected for each antibody. In summary, sensitive detection in bead based flow cytometry has been demonstrated for a large panel of the antibodies and detection of endogenous levels in cultured cells exemplified by Jagged-1 in embryonic stem cells.

### Expression profiling using tissue microarrays

**Figure 6:**
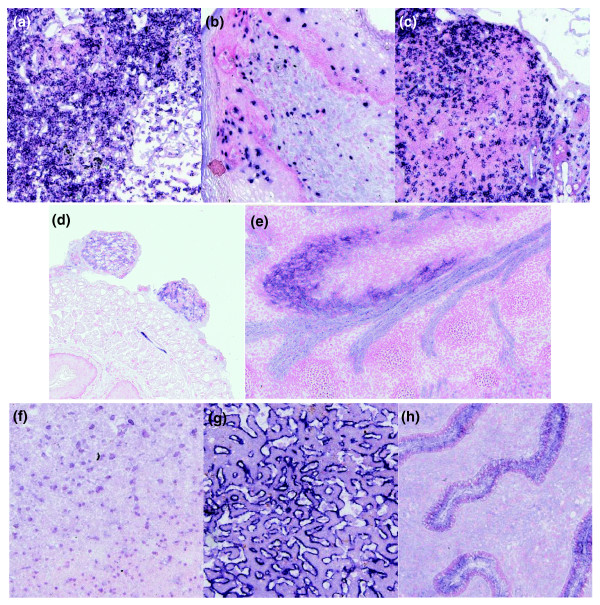
High throughput immunohistochemistry on tissue microarrays. Example data demonstrating cell surface staining of **(a-c) **CD5 (antibody ant165_155_E04) on lymphocytes in human lymphoid tissue, **(d-e) **nerve growth factor receptor (antibody ant54_71_C07) in nerve bundles attached to the murine esophagus and in developing nerve tracts in E14.5 embryo and **(f-h) **nuclear staining of the transcription factor ELF1 (antibody ant46_62_F12) in cerebrum, liver and uterus. Tissues are: (a) lymph node; (b) skin; (c) Payer's patch in small intestine; (d) murine nerve bundles in esophagus; (e) nerve tracts in 14.5 day embryo with myoblast sheet above; (f) cerebrum; (g) liver; and (h) uterus.

We used 381 antibodies that had passed secondary screening to stain acetone fixed frozen tissue microarrays. From this survey, positive staining was observed for 143 (37%) antibodies representing 46/56 antigens tested. Figure 6a-c illustrates an example of an anti-CD5 antibody giving expected staining of scattered human lymphoid cells in lymph node, skin and Peyers patches [16]. We also illustrate immunohistochemical staining with some of the antibodies discussed in previous sections. For example, Figure 4d illustrates detection of endogenous levels of Jagged-1 in the developing kidney of a 14.5 day embryo. This result was confirmed by *in situ *hybridization as shown in Figure 4e. In addition, the three best performing antibodies against nerve growth factor receptor (Ngfr) illustrated in Figure 5 also detected endogenous levels of Ngfr in immunohistochemistry. With these antibodies, expected peripheral nerve staining [17] was present in several adult cores, including large ducts in salivary gland and oesophagus (Figure 6d). Occasional clustered staining was seen in sagittal brain and was also present in a 14.5 day murine embryo where the edge of the central nervous system and spinal cord, choroid, dorsal root ganglia and large nerve tracks were stained strongly (Figure 6e).

Phage display provides the opportunity to generate multiple independent antibodies to provide confirmation of generated expression profiles. Details of overlapping staining profiles with multiple antibodies are given (Additional data file 4). In summary, we demonstrate that it is possible to generate antibodies in high throughput for use in protein expression profiling by immunohistochemistry with an overall success rate consistent with that achieved using hybridoma derived monoclonal antibodies and polyclonal antibodies [3,18,19].

## Discussion

We describe the creation of an antibody phage display library of over 10^10 ^clones and its application to high throughput antibody generation and characterization. Many factors affect success rate throughout the individual steps of the process described here. For example, success rate of protein expression is variable and we have previously described the effect of sequence features and choice of fusion partner in *E. coli *expression [4]. In *E. coli*, receptor extracellular domains were particularly badly expressed, resulting in insoluble inclusion body formation (not shown). This may reflect a requirement for receptors to be trafficked through a eukaryotic secretion system, to ensure correct disulphide bond formation, native protein folding, good secreted protein expression yields and protein stability during purification and storage. In this study we have therefore utilized both bacterial expression and a transient expression system based in mammalian cells [6]. We used the mammalian expression system for receptor extracellular domains, which were the major class of proteins expressed in this system.

Table 1a summarizes the success rate achieved for this class of protein. Approximately 70% of gene constructs gave rise to a soluble product in culture supernatant, as judged by western blot using an anti-His tag antibody (not shown). For 62% of the positive constructs, 50 μg of purified protein were purified from a 50 ml culture, providing sufficient material for antibody generation/screening. The success rate was improved further in some cases by adjusting the boundaries of the expressed domain and by using larger culture volumes (unpublished).

**Table 1:** Success rates in protein expression, antibody isolation and immunohistochemistry

Step	Number	Percentage
**(a) Mammalian expression of receptor extra-cellular domains**		
Sequenced confirmed clones	157	100%
Detected in supernatant by western blot	108	69%
50 μg purified product generated from 50 ml culture	67	62% (43% cumulative)
		
**(b) Success rate of antigens in generation of antibodies**		
Mammalian antigens for selection	205	100%
Antigens generating antibody	181	88%
Bacterial antigens for selection	192	100%
Antigens generating antibody	103	54%
**Total antigens for selection**	**404**	**100%**
**Antigens generating antibody**	**292**	**72%**
		
**(c) Success rate for primary antibody screening**		
Number of antibodies screened	38,164	100%
Number positive on primary screen	9,384	24.6%
Unique clones generated	7,236	77.1% (19% cumulative)
		
**(d) Success rate for secondary antibody screening**		
Clones undergoing secondary specificity screening	4,437	100%
Positive in secondary screening	3,400	76.6%
Specificity demonstrated	2,725	80.1% (61% cumulative)
		
**(e) Success rate in immunohistochemistry**		
Stained on tissue microarrays	381	100%
Positive staining observed	143	37%

The selection success rate was dependent on the antigen source, with mammalian produced antigens being superior to bacterially produced antigens (Figure 2, Table 1b). In mammalian systems, misfolded proteins are degraded [20], and this additional quality control may have led to improved selection results. In contrast, most of the bacterially produced proteins were fused to tags such as maltose binding protein (MBP), which enhanced production of soluble product [4]. Although this has improved the yield of soluble product, it is possible that there is a greater proportion of misfolded protein in the bacterially produced antigen preparations depending on the protein target being expressed [21-23], giving higher backgrounds and compromising selections. For example, expression of the HPV oncoprotein E6 in *E. coli *as an MBP fusion resulted in high soluble expression levels but a major fraction was misfolded aggregate with a minor fraction containing properly folded protein with full biological activity [24].

Where antibodies are raised by selection on native like protein (for example, by phage panning on extracellular domains generated by mammalian expression), it might be expected that many of the antibodies produced will recognize the native form of antigen. Subsequent recognition by such antibodies of both native and denatured forms will then be dependant on whether the target epitope is available after denaturation, for example, within fixed tissues. This is in contrast to other approaches where denatured antigens are used to raise antibodies, for example, by immunization [3,18,19]. Here, recognition of native antigen by antibodies raised on denatured antigen will depend on whether the linear epitope is found in native conditions. Thus, antibodies raised by different approaches may have different utility in recognizing native protein and different sensitivities to fixation/denaturation.

In general terms, we observe a success rate of 37% for generating a positive profile (Table 1d; detailed in Additional data file 3), which is broadly similar to that described previously [3,18,19]. It is difficult, however, to compare immunohistochemistry results in a more specific way across projects since different organisms, genes and antibody generation/fixation regimes are used. In addition, specificity of antibody staining is an important consideration and different groups verify staining patterns to different extents. For example, false positive binding to unrelated antigens can occur, not just with polyclonal antibodies, but also with monoclonal antibodies, as exemplified by previous studies with antibodies on protein microarrays [25-27]. In a similar way, exposing hybridoma derived monoclonal antibodies to the majority of tissue in the body has been shown to result in false positive staining [18]. Systematic verification of staining patterns from expression databases or literature can provide supporting evidence (for example, widespread nuclear expression of ELF1 (Figure 6g-i)). *In situ *hybridization can also provide supporting evidence that a given profile is correct (as exemplified for Jagged-1 in Figure 4d,e). Finally, the availability of multiple antibodies to the same target can increase confidence in the veracity of a given profile by independently generating similar or identical profiles [18]. Among the positive antibodies profiled here, 58 antibodies (15 genes) represented cases where more than one antibody gave the same or overlapping profiles (examples in Figure 6 and summarized in Additional data file 3).

Here, we show the feasibility of antibody generation in a high throughput manner. The bottlenecks in this process was not antibody generation but the cloning, verification and generation of high quality antigen and the preparation and use of the antibodies in downstream applications, for example, in immunohistochemistry. The library and selection process employed was particularly effective, resulting in 7,236 unique antibodies from screening and sequencing 38,164 clones (Table 1c). From 292 successful selections, an average of 25 unique binding antibodies was generated for every 94 clones screened. The success was due to steps taken during library construction to maintain diversity to a maximum and also due to optimization of the selection and screening process (discussed previously in Results). Having developed the library and established the system for carrying out this process, 48-96 antigens can be selected and undergo primary screening and sequencing by 2 people over a 6 week period.

In this study we have sequenced and screened 38,000 antibodies and describe different degrees of characterization for a proportion of these, ranging from specificity ELISA through to detection of endogenous levels of antigens using arrays of adult tissue. A standardized system of validation of antibodies across multiple labs would add to the value of high throughput antibody generation initiatives and the use of antibodies in general. Antibody characterization is complex, however, since the utility of antibodies is application dependent and is subject to many factors, including endogenous levels of antigen expression, availability of epitopes and their sensitivity to fixation or denaturation, antibody affinity/specificities and the sensitivity of detection system used. The affinity of an antibody for a given antigen is a property that can be measured in absolute terms and is related to performance, provided the test antigen accurately represents the target antigen in a biological sample. Affinity determination using surface plasmon resonance provides accurate affinity values but is laborious and requires specialist equipment that is not universally available. Inhibition ELISA can, within limitations, also provide affinity values [14]. In this study we have utilized a simple, sensitive end-point ELISA assay with wide dynamic range to measure binding and permit performance ranking of the clones (following normalization of antibody concentration). We have also introduced a surrogate performance assay for flow cytometry, replacing cells with beads with different antigen coating densities, allowing a measurement of sensitivity in terms of target density per bead. In a similar way, a simple surrogate could be envisioned to rank the potential of clones as western blot reagents by measuring detection limits of denatured antigen on support matrices such as PVDF. While such assays do not guarantee success with biological samples, they can facilitate ranking of clones for subsequent investigations.

Antibody specificity is another important property that relates to the relative affinity for other 'irrelevant' epitopes. Cross-reactivity can arise through conserved interactions with related antigens or by 'polyspecificity' involving different modalities of binding to unrelated antigens [28,29]. It is difficult to be definitive about measuring cross-reactivity since it depends on what antigens are presented in any application. Immunohistochemistry on microarrays of fixed tissues to determine global protein expression pattern represents a particularly challenging application with a high proportion of the proteome being presented at varying levels. Nonetheless, in this study we demonstrate specific detection of non-abundant proteins and provide supporting data from independent antibodies, *in situ *hybridization or alternative data sources to confirm the resultant staining profiles for a proportion of these (Additional data file 3).

To identify obviously cross-reactive clones in a simple 'first pass' assay, we have used a panel of 11 irrelevant antigens, including non-mammalian lysates. Protein microarrays or multiplex bead assays would be more informative in providing a wider range of proteins and these may become more widely available in the future. Standardized western blotting of irrelevant tissue lysates (such as yeast/zebrafish lysates used here) may provide an alternative source of 'arrayed' irrelevant proteins using a technique currently available in many labs that could identify binders with relatively low specificity.

## Conclusion

Historically, antibody validation has been *ad hoc*, making it difficult to compare antibodies from different sources, resulting in much wasted effort and cost. Here we demonstrate the high throughput generation and validation of recombinant antibodies on an unprecedented scale. Large scale efforts to generate and validate antibodies will not only provide a wealth of reagents for research and diagnostics, but could also drive the introduction of a more consistent quality control framework to facilitate comparisons of binders from different sources for use in different applications, whatever the source.

## Materials and methods

### Antigen cloning and expression

Antigens were obtained either from commercial sources, or were expressed from *E. coli *or transient mammalian expression systems using systems we have described previously [30]. For primer design, mRNA sequence was downloaded from the Ensembl entry [31] with coding and 5' and 3' untranslated region (UTR) annotation. Pfam domains [30] were annotated onto the nucleic acid sequence and primers designed to amplify either the full-length open reading frame or specific Pfam domains. Inserts were amplified from cDNA using nested PCR, as described previously [4], GATEWAY cloned into expression vectors [32] and DNA sequence confirmed. All steps in antigen production, including PCR amplification, sequence confirmation, expression and purification, were electronically tracked using a custom designed laboratory information management system (unpublished). In the case of automated primer design of the immunoglobulin superfamily (IgSF), targets were identified from a search of the ENSEMBL database (NCBI m34 mouse assembly) for ENSMUSP (protein) entries that contained annotated IgSF Pfam domains and signal peptides. The transcript sequence with annotated protein features, including coding sequence signal peptides, transmembrane (TM) regions and Pfam domain boundaries, were batch downloaded and used as a template for automated primer design. Outer primers were designed to the UTRs to amplify the full-length open reading frame using Primer3 [33]. Three design strategies were employed for the inner primers: EC+SIGP-euk, coordinates 1 to minus 5 amino acids upstream of the TM, minus a stop codon for HEK293E expression with carboxy-terminal tags; EC-SIGP-pro, coordinates plus 1 amino acid downstream of the SIGP to minus 5 amino acids upstream of the TM plus a stop codon for *E. coli *expression with amino-terminal tags; and IC-pro, coordinates plus 5 amino acids downstream of the TM to the end of the coding sequence plus a stop codon for *E. coli *expression with amino-terminal tags.

Our *E. coli *protein expression system was described previously [4] and utilizes the T7 RNA polymerase promoter driving cytoplasmic expression in BL21-DE3 cells (Novagen, Nottingham, UK). This line has an inducible RNA polymerase gene under the control of the lac promoter. Expression was performed using either shake flasks or 24-well blocks with the Studier auto-induction protocols [34]. Most proteins were expressed with an amino-terminal decahistidine (His10) tag for affinity purification and either a thioredoxin (Trx) or MBP solubility enhancing fusion. In common with many low molecular weight eukaryotic proteins [4], the Rab proteins did not require a solubility enhancing fusion for high level soluble expression. Expression in mammalian cells was by transient transfection of HEK293E suspension cells [6] with expression vectors designed to add a carboxy-terminal His10 tag or His10-rat Cd4 (domains 3 and 4) fusion [35]. Most receptor ectodomains were expressed in HEK293E cells at a 50 ml culture volume, but low expressing clones were scaled up to a 200 ml culture volume. Proteins were affinity purified using a FPLC system or Qiagen8000 robot. All proteins were subject to a quality control procedure before handover for antibody selection, which required a minimum of 50 μg and greater than 80% purity of full-length target protein. Proteins were stored in a buffer containing 50% or 10% glycerol for -20°C or -80°C storage, respectively

### Construction of pSANG4 display vector

A modified version of the phage display vector pHEN1 [36] was constructed by inserting a novel/cloning linker region. This was created by annealing primers NcNotlinkS and NcNotlinkA, extending with DNA polymerase and cutting with *Nco*I/*Not*I before cloning into the *Nco*I/*Not*I site of pHEN1 to give pSANG3. This allows sequential cloning of antibody light chains via *Nhe*I/*Not*I and heavy chains via *Nco*I/*Xho*I. The *pelB *signal sequence in pSANG3 was replaced with the signal sequence from M13 gene 3 to create the vector pSANG4. This leader is potentially more useful for ligation independent cloning. (Ligation independent cloning allows direction cloning without restriction enzymes or ligase enzymes, by using the 5'-3' exonuclease activity of DNA polymerase to create single stranded overhangs for annealing. The presence of a single nucleotide causes the exonuclease activity to arrest where that nucleotide becomes incorporated and so long overhangs can be engineered [37].) The M13 leader and 5' UTR were created by annealing oligonucleotides G3HindNdeS and G3NcoA (see Additional data file 4), extending with DNA polymerase (Novagen), digesting and cloning into the *Hin*dIII and *Nco*I sites of pSANG3. G3HindNdeS is based on the sequence of M13 but adds a stop codon to stop elongation initiated at the *lac*Z start codon, which is upstream in the vector. The primers also introduce a new *Nde*I restriction site at the start of the leader sequence and introduce a silent mutation in the tenth codon of the leader with a view to increasing the potential overhang in ligation independent cloning. Constructs were confirmed by sequencing with primers LMB3 and fdtseq1. The resultant vector, pSANG4, is represented in Figure 1 and more fully described in Additional data file 4.

### Construction of display library

An antibody phage display library was created by sequentially cloning a repertoire of light chain variable regions (VL) followed by cloning of heavy chain variable regions (VH) in pSANG4. The VH and VL pools were cloned through primer encoded *Nco*I/*Xho*I and *Nhe*I/*Not*I sites, respectively. The VH and VL domains were initially cloned into an intermediate, out of frame cloning vector, pSANG2. The final format of the antibody is a scFv with VH and VL fragments joined by a flexible linker peptide (Gly_4 _Ser Gly_4 _Ser Gly_3 _Ala Ser).

A more detailed description of the library construction is provided in Additional data file 4. In brief, antibody heavy and light chain variable region repertoires were created by PCR amplification from human lymphocytes. Fourteen heavy and 28 light chain primer pairs were used and maintained separately in 462 separate primary PCRs with cDNA originating from 43 lymphocyte donors. Light chains were cloned as *Nhe*I/*Not*I fragments. To maintain diversity, the VL repertoire was cloned as 9 separate sub-libraries with an average of 10^7 ^clones per library (6 kappa families and 3 pools of lambda families). Plasmid DNA was prepared from these and pooled into two separate kappa or lambda chain sets. The VH repertoire was sub-cloned via *Nco*I*/Xho*I sites to these two light chain libraries. Fourteen different sub-libraries were prepared, consisting of each of the seven heavy chain families (VH1-VH7) combined with either kappa or lambda light chains. It has been suggested [13] that particular combinations of VH and VL families express better and so two additional sub-libraries of VH3/κ3 and VH3/κ1 were generated, giving a combined total of 1.1 × 10^10 ^inserts in the 'McCafferty' phage display library.

### High throughput selection

Rescue of phage particles from the library was carried out essentially as described [38] but using trypsin-cleavable helper phage [11,39], which increased the efficiency of binder isolation such that only two rounds of selection were required. Selection was as described previously [38] with the following modifications (described in more detail in Additional data file 4). Following the rescue of the individual library aliquots, the antibody-phage particles were purified by sequential polyethylene glycol precipitation (Sigma, Poole, UK) and cesium chloride gradient centrifugation and the quality assessed by confirming the presence of full length antibody-gene 3 fusion by western blotting using anti-gene 3 antibody (New England Biolabs, Hitchin, UK; data not shown). Each aliquot was titrated and pooled according to the size of the aliquot to normalize the contribution of each clone. Final concentration was 10^13 ^colony forming units/ml and 50 μl was used per selection. For the selections, each antigen was coated at 10 μg/ml onto a single well of a 96-well microtiter plate. We routinely performed a deselection process before commencing the first round of selection for recombinant protein targets with common fusion partners, for example, human IgG1 Fc, rat CD4 (domains 3 and 4), MBP, and Trx. This procedure helped to deplete binders to fusion partners.

Two rounds of selections were performed using the 'McCafferty' scFv antibody-phage library. Following binding of the library to immobilized antigen, phage were eluted in 100 μl of freshly prepared 125 μg/ml TPCK-trypsin (Sigma) in 50 mM Tris pH 8, 1 mM CaCl_2 _at room temperature for 15 minutes. A mid-log culture of *E. coli *TG1 cells (200 μl) was added to each well containing the eluted phage and shaken slowly (150 rpm) at 37°C for 1 hour. At this stage aliquots can be taken to titer the output. Bacteria were pelleted at 3,000 × g for 10 minutes, resuspended in 500 μl of 2 × TY medium with ampicillin and 2% glucose (2 × TY amp/glu) and grown overnight at 30°C. The next day, 1 ml cultures of 2 × TY amp/glu in a deep well plate were inoculated with 20 μl of the overnight culture and incubated at 37°C for 1 hour at 600 rpm. Helper phage were added to a MOI of 10 and incubated with gentle shaking at 150 rpm at 37°C for 1 hour. Bacteria were pelleted at 3,000 × g for 10 minutes, resuspended in 500 μl 2 × TY medium with ampicillin and kanamycin and incubated overnight at 30°C with shaking at 600 rpm. The selected outputs were then evaluated for specific antigen binding using phage ELISA [40]. Individual, selected scFv colonies were rescued as scFv-phage particles in 96-well microtitre wells and transferred to specific antigen (5 μg/ml) coated wells. Anti-M13-horseradish peroxidase conjugate (GE Healthcare, Little Chalfont, UK) and 3,3',5,5'-tetramethyl benzidine substrate (Sigma) were used to detect bound phage.

### Sub-cloning selected populations

Prior to screening of individual clones, the selected populations were sub-cloned into an expression vector pSANG14-3F [7]. This vector fuses the antibody to alkaline phosphatase to drive dimerization and facilitate production of antibody product and subsequent detection of binding. It also introduces a hexahistidine tag for purification and a tri-FLAG tag for detection of binding via secondary anti-FLAG antibodies (Sigma). Sub-cloning of selected populations prior to screening also increased success rate by reducing the proportion of growth-advantaged clones with truncated inserts in the screened population. Selected antibody gene populations were amplified by PCR from the glycerol stocks of the second round of selection using M13 LeadSeq (5'-AAATTATTATTCGCAATTCCTTTGGTTGTTCCT-3') and NotMycSeq (5'-GGCCCCATTCAGATCCTCTTCTGAGATGAG-3') primers. The amplified DNA was run on a 1% agarose gel; insert excised and purified using a QiaQuick PCR purification kit (Qiagen, Crawley, UK). Two micrograms of the purified PCR products were digested with *Nco*I and *Not*I restriction enzymes (New England Biolabs) and electrophoresed in a 1% agarose/TBE gel. The DNA was purified from the gel slice using the QiaQuick gel purification kit (Qiagen) and then ligated into the *Nco*I/*Not*I digested pSANG14-3F expression vector using T4 DNA ligase (New England Biolabs). After purification of the DNA from the ligation reaction, the ligated product was electroporated into electrocompetent BL21 (DE3) cells (Novagen). Ninety-four clones per target were picked, grown and stored at -80°C.

### Primary screening of selected populations

The primary and secondary screening ELISAs were performed essentially as described previously [7]. All primary screening ELISAs were performed on scFv released from bacterial pellets, following overnight induction, using Bug Buster lysis buffer (Novagen). For the primary screening ELISA, we initially performed the screening assay in a 96-well format using direct detection of scFv binding via the alkaline phosphatase fusion protein and addition of pNPP substrate (Sigma). For each target, one 96-well flat bottomed Maxisorp polystyrene plate (Nunc, Roskilde, DK) was coated with the specific antigen and one ELISA plate with the appropriate control antigen (for example, MBP, Fc, Trx). The scFvs were assayed as single points on each plate and any scFv with a specific antigen signal ≥3-fold higher than the control was scored as a positive. Later assays were performed in 384-well ELISA plates using time resolved fluorescence, which involves using europium labeled secondary detection (Perkin Elmer, Beaconsfield, UK) to mirror the secondary screening assay. Here, for each target, one 384-well flat bottomed black polystyrene plate (Nunc Maxisorp) was coated with both the specific antigen and with the appropriate control antigen. ScFv binding was detected using an anti-FLAG secondary antibody (Sigma) conjugated to europium (Perkin Elmer). Again, an antibody clone was deemed to be antigen specific in the primary screening ELISA where the mean of the two replicate scores on the target antigen was at least three-fold greater than the mean of the two replicate scores for the control protein.

### Secondary screening of selected populations

The secondary screening ELISA was used to identify cross-reactive antibodies. ScFv was produced by overnight growth in 1 ml of auto-induction media [34] in a 96-well deep well microtitre plate (Costar, Lowell, MA, USA). Periplasmic extracts was prepared by centrifugation at 3,000 g for 10 minutes followed by resuspension in 40 μl TES buffer (50 mM TrisHCl, pH 8.0, 1 mM EDTA, 20% (w/v) sucrose, 2 μl/ml protease inhibitor cocktail set VII (CalBioChem, Nottingham, UK)). After 5-10 minutes 60 μl of a one-fifth dilution of TES supplemented with 2.5 mM MgCl, 2 μl/ml protease inhibitor cocktail set VII, and 25 U/ml Benzonase (VWR, Lutterworth, UK) were added. Recombinant antibody fused to alkaline phosphatase (scFv-AP) was mixed with phosphate-buffered saline (PBS)/1% milk protein then transferred to pre-blocked 384-well assay plates for development of the ELISA. For convenience, the scFv-AP-antigen binding step was performed overnight at 4°C. Six 384-well ELISA plates were required to screen each plate of clones. There were two antigens per 384-well plate coated in duplicate for the panel of 12 antigens. The antigen panel comprised the specific antigen, a fusion partner control, keyhole limpet hemocyanin, thyroglobulin, myoglobin, cytochrome c, human IgG, laminin, fibronectin, α-glycerol phosphate dehydrogenase (Sigma) and total protein lysates from zebra fish (*Danio rerio*) and yeast (*Schizosaccharomyces pombe*). Plates were washed and bound scFv-AP detected with an anti-FLAG secondary antibody conjugated to biotin (Sigma) followed by a streptavidin-europium conjugate (Perkin Elmer), which was detected using time resolved fluorescence.

### Sequence analysis

Each plate of 94 scFv clones, corresponding to a single target antigen, was sequenced using a single forward primer, pSANG Fwd (5'-TATGAAATACCTGCTGCCGACC-3'). Sequences were analyzed using Blaze 2, software from Cambridge Antibody Technology (Cambridge, UK). Following alignment, duplicate clones were identified and removed. The remaining scFv clones were then aligned according to the sequence of the heavy chain CDR3 region, which is the most variable segment. Up to 22 clones per target were chosen based on both their signal-to-noise ranking from the primary screening ELISA (highest to lowest) and on the diversity of their HCDR3 amino acid sequences. Selected clones were 'cherry picked' for further sequence and specificity analysis, with each derivative plate containing four sets of scFv specific to four different target antigens. The selected scFv clones were sequenced in depth using six sequencing primers, three forward primers (VF1: 5'-GGGGAATTGTGAGCGGA-3'; VF2: 5'-GATCGAGATCTCGATCCCGCGA-3'; Hlink: 5'-ACCGCCAGAGCCACCTCCGCC-3') and three reverse primers (Llink: 5'-GGCGGAGGTGGCTCTGGCGGT-3'; VR1: 5'-CGTGCGGCAGTAATTTCC-3'; VR2: 5'-TGTAGTAATATCGCCCTGAGCAGCC-3'). The reads were assembled using phrap, and a minimum read depth of 3 was required for all bases before any consensus call was accepted. The consensus DNA sequence was subjected to six frame translation and vector encoded sequences flanking the VH and VL genes identified (the *pelB *signal sequence, the scFv linker sequence (Gly_4 _Ser Gly_4 _Ser Gly_3 _Ala Ser) and the alkaline phosphatase gene). The closest germline variable region gene was identified and the various framework and CDRs were identified.

### Performance ranking of scFvs using flow cytometry calibration beads

Flow cytometry calibration beads with defined numbers of anti-human IgG receptors (Quantum Simply Cellular anti-Human IgG bead sets, Bangs Laboratories Inc., Fishers, IN, USA)) were used to assess antibody performance in flow cytometry. This allows the generation of beads with defined amounts of human Fc fused antigens, permitting an assessment of sensitivity limits for selected antibodies. For example, the set described in Figure 4 used beads with 29,588, 83,460, 204,228, and 618,888 antigen copies per bead. Beads were incubated with blocking buffer (PBS/0.1% (w/v) bovine serum albumin (BSA; Sigma)/0.01% (w:v) NaN_3_) prior to incubation with an excess of Fc-tagged antigen. Following binding of the antigen, the beads were washed by centrifugation in blocking buffer (300 × g for 4 minutes), and blank (uncoated) beads were then added to the pool of beads. The final bead mixture was dispensed into 96-well plates. Following a further wash step, as above, 100 μl of scFv at 2 μg/ml or a suitable control was added to the beads and allowed to bind for 1 hour on ice. After washing, bound scFv was detected using anti-FLAG-biotin (Sigma) at 1 μg/ml followed by streptavidin-PE (Pharmingen, Oxford, UK) at 0.5 μg/ml. Antigen binding to the beads was confirmed by staining with an Fc-specific anti-IgG-FITC antibody (Jackson Immunolaboratories, West Grove, PA, USA). This antibody did not bind to the beads in the absence of Fc-tagged antigen. Data were collected using a FC500MPL flow cytometer (Beckman Coulter, High Wycombe, UK), WinMDI [41] was used to create the histogram plots shown in Figure 4, and median fluorescence intensities of each of the bead peaks were generated using the log/log method within the CXP software (Coulter). The limit of sensitivity for each scFv clone was determined using the QuickCal v2.3 software (Bangs Laboratories).

### Flow cytometry with ES cells

46C mouse embryonic stem cells were grown in LIF supplemented medium in feeder-free adherent culture as described [42]. This cell line is known to express Jagged-1 [15,42]. Cells were removed from adherent culture using trypsin EDTA and stained in 96 U-well plates in 100 μl at 3 × 10^5^/well with all procedures being performed on ice. ScFv were applied for 1 hour at 1 μg/ml diluted in PBS supplemented with 1% BSA and 0.01% sodium azide. Following washing, anti-FLAG-biotin (Sigma; clone M2) was applied at 1 μg/ml diluted in PBS supplemented with 0.1% BSA and 0.01% sodium azide. After an hour's incubation, the cells were washed and 100 μl of streptavidin-phycoerythrin conjugate (Becton Dickinson, Oxford, UK) was applied at 0.5 μg/ml for 30-60 minutes. After washing, the cells were resuspended in PBS/0.1% BSA/sodium azide and staining was visualized using a FC500-MPL flow cytometer. Analysis was performed using WinMDI with dead cells gated out as determined by forward versus side scatter profiles. No staining was evident in the absence of scFv or in the presence of an irrelevant scFv. Histogram plots showing levels of staining with streptavidin-phycoerythrin for control and test are shown.

### Immunohistochemistry

Frozen tissue arrays of normal adult human tissue and developmental and adult murine tissue were prepared by Covance Ltd (Yorkshire, UK) as previously described by Kononen *et al*. [43]. These encompassed one slide type of 28 different human adult tissues or a second slide type of 23 different adult murine cores, with murine adult sagittal brain and E14.5 embryo sections added. In each array, two tissue cores were included from each donor sample and two donors were used to represent each tissue type. Sections of the microarrays were fixed by immersion in acetone at ambient temperature for 15 minutes, blocked for endogenous alkaline phosphatase activity (DAKO, Ely, UK) and non-specific protein interaction. Alkaline phosphatase-fused scFv antibodies were incubated for 1 hour in blocking solution then an alkaline phosphatase-labeled anti FLAG tag antibody (Sigma) was applied for 30 minutes to amplify the quantity of label for enzyme detection using NBT/BCIP substrate (DAKO), which was also applied for 30 minutes. Although binding can be detected directly via the alkaline phosphatase fusion partner, we have found improved sensitivity using a secondary alkaline phosphatase labeled antibody [7]. The arrays were counterstained with nuclear fast red (Vector Labs, Peterborough, UK), dehydrated in alcohol, cleared in xylene and mounted in Eukitt resinous mountant (ProSciTech, Poway, CA, USA)).

### *In situ *hybridization

E14.5 mouse embryos (C57BL/6JTyrC-Brd) were fixed in 10% neutral-buffered formalin for 48 hours and embedded in paraffin wax. Eight micrometer sections on super frost + slides were hybridized with a 1,170 bp digoxygenin-labeled antisense RNA probe to the mouse Jag1 gene (ENSMUSG00000027276) generated from a DNA template. The template was synthesized by RT-PCR with gene specific primers (Jag1_1_f: CTAGGCCTGGAGCTTCCACATCTGC; Jag1_1_r: TAATACGACTCACTATAGGGAGCAGTCCCGGTGGTGAACCTGGAT).

Transcription was performed using an Ambion Maxi-Script kit with the addition of digoxygenin UTP (Roche, London, UK). Sixty nanograms of probe were used for hybridization at 65°C for 6 hours, using a Ventana (Illkirch, France) Discovery platform with BlueMap and RiboMap kits, according to manufacturer's guidelines. The final stringency of washing was 3 × 0.1 SSC at 85°C. Sections were counterstained with Nuclear Fast Red

## Abbreviations

BSA, bovine serum albumin; CDR, complementarity determining region; ES, embryonic stem; MBP, maltose binding protein; Ngfr, nerve growth factor receptor; PBS, phosphate-buffered saline; scFV, single chain Fv; TM, transmembrane; Trx, thioredoxin; UTR, untranslated region; VH, heavy chain variable region; VL, light chain variable region.

## Authors' contributions

JMC coordinated and led the overall project, designed and helped construct the library (with MOS and KJV) and prepared the manuscript along with DJS. Protein production from primer design and gene cloning through to protein purification and quality control was led by MRD and carried out by MRD, SDJC, AMC, RLP, SPS. DJS led the selection and screening of the antibody library along with ARP, VC, SK, CDM and WR. WEW prepared antibodies for immunohistochemistry and flow cytometry. JLY/ARP carried out the flow cytometry work and JLY characterized antibodies to Notch and jagged antigens. Immunohistochemistry was carried out by KFC, JSC, GF, YH, WJH, and JNM, led by AW. AKK carried out *in situ *hybridization. Informatics support, including sequence analysis and database/website construction, was provided by SREC, GJG, GLM, and JX.

## Additional data files

The following additional data are available with the online version of this paper. Additional data file 1 is a table listing all 406 antigens used in antibody selection and the number of unique antibodies generated. Additional data file 2 is a table listing median fluorescent intensities of antigen beads as plotted in Figure 5. Additional data file 3 provides a description of staining profiles supported by multiple antibodies in immunohistochemistry. Additional data file 4 describes the construction and rescue of the 'McCafferty' antibody phage display library.

## Supplementary Material

Additional data file 1 All 406 antigens used in antibody selection and the number of unique antibodies generated. Click here for file

Additional data file 2 Median fluorescent intensities of antigen beads as plotted in Figure 5. Click here for file

Additional data file 3 Description of staining profiles supported by multiple antibodies in immunohistochemistry. Click here for file

Additional data file 4 Construction and rescue of the 'McCafferty' antibody phage display library Click here for file
