# Supplementary material for: Application of phage display to high throughput antibody generation and characterization
Source: Genome Biol. 2007 Nov 29;8(11):R254. doi: 10.1186/gb-2007-8-11-r254 (PMC2258204; doi:10.1186/gb-2007-8-11-r254)
Supplement: Additional data file 1 — All 406 antigens used in antibody selection and the number of unique antibodies generated. [file gb-2007-8-11-r254-S1.doc]

**Additional data file 1. Supplementary Methods**

**Construction and rescue of the “McCafferty” antibody phage display library.**

An antibody phage display library was created by sequentially cloning a repertoire of light chain variable regions (VL) followed by cloning of heavy chain variable regions (VH). The heavy and light chain repertoires were created by PCR amplification from human lymphocytes mainly collected from peripheral blood. The VL repertoire was cloned into the *Nhe*I/*Not*I sites of pSANG4. Plasmid DNA was prepared from this library before cloning the VH repertoire into the *Nco*I/*Xho*I sites. The final format of the antibody is a single chain Fv (scFv) with VH and VL fragments joined by a flexible linker peptide (gly_4_ ser gly_4_ ser gly_3_ ala ser).

Diversity was maintained at a maximum by:

1. using donations from 42 peripheral blood donations and 1 tonsil
2. maintaining all donations separately and combining to 11 pools at mRNA stage
3. maintaining separate V region primers (14 heavy chain, 13 kappa light chain and 15 lambda light chains) during primary PCR for each of the 11 pools i.e. 462 separate primary PCR reactions.
4. transforming each of the 6 kappa light chain families separately and pooling the 15 lambda chain families into 3, also transformed separately. These were only pooled into 2 master pools (kappa and lambda) once DNA was isolated from each stock to prepare vector for heavy chain cloning.
5. transforming of each of the 7 heavy chain families individually into both the kappa and lambda light chain libraries creating a total of 14 pools of libraries, supplemented with 2 additional sub-libraries representing VH3/Vkappa1 and VH/kappa3 combinations.

**Construction of pSANG4 vector.** A modified version of the phage display vector pHEN1^36^ was constructed to allow sequential cloning of antibody heavy chains (via *Nco*I and *Xho*) and light chains (via *Nhe*I and *Not*I). pHEN1 was digested with *Nco*I and *Not*I and was treated with Antarctic phosphatase (NEB). A novel/cloning linker region was created by annealing primers NcNotlinkS and NcNotlinkA, extending with DNA polymerase and cutting with *Nco*I/*Not*I. The region of hybridization is shown underlined in the sequence given below. Vector and insert were ligated and transformed into DH5α. Sequencing data of the resultant vector pSANG3 was as expected.

NcNotlinkS:

GCCCAGCCGGCCATGGCCCAGGTGCAGCTGCTCGAGGGTGGAGGCGGTTCAGGCGGAGGTGGCTCT

NcNotlinkA:

TTTTTGTTCTGCGGCCGCGTCATCAGATCTGCCGCTAGCGCCACCGCCAGAGCCACCTCCGCCTGAACC

pSANG4 replaces the pelB signal sequence of pHEN1/pSANG3 with the signal sequence from M13 gene 3. This leader is potentially more useful for ligation independent cloning (LIC). LIC which creates long single stranded overhangs and requires nucleotide stretches which use only 3 of the 4 bases (e.g. e.g. ref 37). The M13 leader and 5’ UTR was created by annealing oligos G3HindNdeS and G3NcoA, extending with DNA polymerase, digesting and cloning into the Hind3 and *Nco*I sites of pSANG3. The region of hybridization is underlined in the sequence given below. G3HindNdeS is based on the sequence of M13 but adds a stop codon to stop elongation initiated at the lacZ start codon which is upstream in the vector. The primers also introduce a new *Nde*I restriction site at the start of the leader sequence (found in vectors such as pET26 vectors) and introduce a silent mutation in the 10th codon of the leader with a view to increasing the potential overhang in ligation independent cloning.

G3HindNdeS

TGATTACGCCAAGCTTTTAGGAGCCTTTTTTTTTGGAGATTTTCAACcataTGAAAAAATTATTATTCGCAATT

G3NcoA

CTGCACCTGGGCCATGGCCGGCTGGGCCGCATAGAAAGGAACAACCAAAGGAATTGCGAATAATAATTTTTTCA

Constructs were confirmed by sequencing with primers LMB3 and fdtseq1

LMB3: CAGGAAACAGCTATGAC

FDTSEQ1: GTCGTCTTTCCAGACGTTAGT

Sequence of the resultant vector is represented in Figure S1.

**Figure S1. Features of phage display vector pSANG4** Sequence of pSANG4 is given between sites for sequencing primers LMB3 and FDTSEQ1, which are shown underlined. The upstream initiation codon from lacZ is shown in italics within the LMB3 primer. Unique restriction sites *Hind*III, *Nde*I, *Nco*I, *Xho*I, *Nhe*I and *Not*I are shown underlined. The amino acid sequences of the leader, linker, myc tag and gene 3 are also shown under the nucleotide sequence.

CAG GAA ACA GCT *ATG* ACC ATG ATT ACG CCA AGC TTT TAG GAG CCT TTT

LMB3 primer Hind III

TTT TTG GAG ATT TTC AAC CAT ATG AAA AAA TTA TTA TTC GCA ATT CCT

Nde1 M K K L L F A I P

___­­______M13 LEADER ____________

TTG GTT GTT CCT TTC TAT GCG GCC CAG CCG GCC ATG GCC CAG GTG CAG

L V V P F Y A A Q P A M A

_____________________M13 LEADER _____________Nco1__

CTG CTC GAG GGT GGA GGC GGT TCA GGC GGA GGT GGC TCT GGC GGT GGC

L E G G G G S G G G G S G G G>

Xho1 _______________________LINKER______________________

GCT AGC GGC AGA TCT GAT GAC GCG GCC GCA GAA CAA AAA CTC ATC TCA

A S A A A E Q K L I S

Nhe1 Not1 ______MYC TAG_________

GAA GAG GAT CTG AAT GGG GCC GCA TAG ACT GTT GAA AGT TGT TTA GCA

E E D L N * T V E S C L A

_____MYC TAG______ amber __________GENE 3__________

AAA CCC CAT ACA GAA AAT TCA TTT ACT AAC GTC TGG AAA GAC GAC AAA

K P H T E N S F T N V W K D D K

FDTSEQ1

**Lymphocyte isolation from peripheral blood.** Lymphocytes were prepared by Ficol gradient centrifugation from buffy coat donations (obtained under Local Research Ethical Committee approval). An average of 5 x 10^8^ cells were obtained from each preparation. Cells were resuspended in *RNAlater* solution (Invitrogen) which stabilizes the RNA, and then stored at 4ºC. RNA was prepared using Trizol (Invitrogen). Tonsil total RNA was isolated using RNAlater followed by “RNeasy maxi” kit (Qiagen). mRNA was prepared using “Oligotex mRNA spin-column” kit (Qiagen). mRNA was used to generate cDNA with a First-Strand cDNA synthesis kit (Amersham) using antibody primers based in the constant region. The primers used for kappa, lambda light chains and IgM heavy chains (previously shown to give a more diverse repertoire^43^ were:

1. Heavy chain: HuIgMFOR TGGAAGAGGCACGTTCTTTTCTTT
2. Kappa light chain: HuGkFOR AGACTCTCCCCTGTTGAAGCTCTT
3. Lambda light chain: HuCLFOR TGAAGATTCTGTAGGGGCCACTGTCTT

**Primary heavy and light chain PCRs.** Antibody genes were amplified from the cDNA using the primers shown in Table S1. To maintain diversity, 40 ng of cDNA (equivalent to average of 7 x 10^5^ cell equivalents) was used in each individual PCR reaction. Primers for amplifying the heavy and light antibody regions were taken from VBASE (http://vbase.mrc-cpe.cam.ac.uk/) and are summarized in Tables S1-3. The J region primers were those that were originally used by Marks *et al*., (1991)^43^. The scheme for PCR amplification and construction of the library is summarized in Figure S2.

**Figure S2. PCR amplification and construction of the single chain Fv (scFv) phage display library**

VkBAK x13

VLBAK x15

JKFOR x 5

JLFOR x 3

VH

VHBAK x14

JHFOR x 4

VL

VH

NcoI

XhoI1

NheI1

NotI1

pSANG4 vector

Light chain library

scFv phage display library

NheI/NotI digest

NcoI/XhoI digest

Antibody Heavy chain cDNA pool

Antibody light chain cDNA pool

PCR

PCR

PCR

PCR

VH

VL

linker

myc

gene 3

leader

NcoI

NotI1

VL

**PCR amplification of heavy chain repertoire.** 14 VH primers, 13 Vk primers and 15 Vl primers were synthesized. These were used individually in PCR reactions, with a second primer pool made up of the 4 JHFOR primers for VH amplification and 5 JKFOR or 3 JLFOR primers for VL kappa and VL lambda light chain amplification respectively (shown in Tables S1, S2).

**Table S1. Sequence of primers used in PCR of heavy chain variable region (VH)**

| Name | Sequence (5’-3’) |
| --- | --- |
| VH1aBAK | CAGGTKCAGCTGGTGCAG |
| VH1bBAK | CAGGTCCAGCTTGTGCAG |
| VH1cBAK | SAGGTCCAGCTGGTACAG |
| VH1dBAK | CARATGCAGCTGGTGCAG |
| VH2aBAK | CAGATCACCTTGAAGGAG |
| VH2bBAK | CAGGTCACCTTGARGGAG |
| VH3aBAK | GARGTGCAGCTGGTGGAG |
| VH3bBAK | CAGGTGCAGCTGGTGGAG |
| VH3cBAK | GAGGTGCAGCTGTTGGAG |
| VH4aBAK | CAGSTGCAGCTGCAGGAG |
| VH4bBAK | CAGGTGCAGCTACAGCAG |
| VH5aBAK | GARGTGCAGCTGGTGCAG |
| VH6aBAK | CAGGTACAGCTGCAGCAG |
| VH7aBAK | CAGGTSCAGCTGGTGCAA |
| JH1-2FOR | TGAGGAGACGGTGACCAGGGTGCC |
| JH3FOR | TGAAGAGACGGTGACCATTGTCCC |
| JH4-5FOR | TGAGGAGACGGTGACCAGGGTTCC |
| JH6FOR | TGAGGAGACGGTGACCGTGGTCCC |

Ambiguity codes M=A/C, R=A/G, S=G/C, W= A/T

An additional set of 14 VHBAK primers and 4 JHFOR primers with a 5’ extension was prepared to introduce *Nco*I/SfiI and *Xho*I at the 5’ and 3’ end respectively of the primary VH PCR product. A comparison of the original and the extended primers is shown below with the extension sequence shown in lower case and restriction sites underlined.

VH1aBAK CAGGTKCAGCTGGTGCAG

VH1aBAKSfi gtcctcgcaactgcggcccagccggccatggccCAGGTKCAGCTGGTGCAG

JH1-2FOR TGAGGAGACGGTGACCAGGGTGCC

JH1-2FORXho tgaaccgcctccaccactcgagTGAGGAGACGGTGACCAGGGTGCC

**Table S2. Sequence of primers used in primary PCR of light chain variable region (VL)**

| Vk1aBAK | RACATCCAGATGACCCAG |
| --- | --- |
| Vk1bBAK | GMCATCCAGTTGACCCAG |
| Vk1cBAK | GCCATCCRGATGACCCAG |
| Vk1dBAK | GTCATCTGGATGACCCAG |
| Vk2aBAK | GATATTGTGATGACCCAG |
| Vk2bBAK | GATRTTGTGATGACTCAG |
| Vk3aBAK | GAAATTGTGTTGACRCAG |
| Vk3bBAK | GAAATAGTGATGACGCAG |
| Vk3cBAK | GAAATTGTAATGACACAG |
| Vk4aBAK | GACATCGTGATGACCCAG |
| Vk5aBAK | GAAACGACACTCACGCAG |
| Vk6aBAK | GAAATTGTGCTGACTCAG |
| Vk6bBAK | GATGTTGTGATGACACAG |
| VL1aBAK | CAGTCTGTGCTGACTCAG |
| VL1bBAK | CAGTCTGTGYTGACGCAG |
| VL1cBAK | CAGTCTGTCGTGACGCAG |
| VL2BAK | CAGTCTGCCCTGACTCAG |
| VL3aBAK | TCCTATGWGCTGACTCAG |
| VL3bBAK | TCCTATGAGCTGACACAG |
| VL3cBAK | TCTTCTGAGCTGACTCAG |
| VL3dBAK | TCCTATGAGCTGATGCAG |
| VL4BAK | CAGCYTGTGCTGACTCAA |
| VL5BAK | CAGSCTGTGCTGACTCAG |
| VL6BAK | AATTTTATGCTGACTCAG |
| VL7BAK | CAGRCTGTGGTGACTCAG |
| VL8BAK | CAGACTGTGGTGACCCAG |
| VL4/9BAK | CWGCCTGTGCTGACTCAG |
| VL10BAK | CAGGCAGGGCTGACTCAG |
| JK1FOR | ACGTTTGATTTCCACCTTGGTCCC |
| JK2FOR | ACGTTTGATCTCCAGCTTGGTCCC |
| JK3FOR | ACGTTTGATATCCACTTTGGTCCC |
| JK4FOR | ACGTTTGATCTCCACCTTGGTCCC |
| JK5FOR | ACGTTTAATCTCCAGTCGTGTCCC |
| JL1FOR | ACCTAGGACGGTGACCTTGGTCCC |
| JL2-3FOR | ACCTAGGACGGTCAGCTTGGTCCC |
| JL4-5FOR | ACCTAAAACGGTGAGCTGGGTCCC |

Ambiguity codes M=A/C, R=A/G, S=G/C, W= A/T

For addition of restriction sites to the VL products, an additional set of primers was synthesized with a 5’ extension added to the VKBAK and VLBAK primers to introduce an *Nhe*I restriction site. To reduce the number of secondary PCRs primers, VKBAKNhe and VLBAKNhe primers were designed based on similar families, reducing the range of VκBAKNhe primers from 13 used in the primary PCR set to 3 used in the secondary PCR and the number of VlBAKNhe primers from 15 used in the primary PCR set to 3 used in the secondary PCR (Table S3). Thus Vk1aBAK derived primary PCR products are amplified by VkBAKNheA as shown, with the extension sequence in lower case and the *Nhe*I site underlined:

Vk1aBAK RACATCCAGATGACCCAG

VkBAKNheA tctggcggtggcgctagcRACATCCAGATGACCCAG

For the JKFOR and JLFOR primers, an extension was added to the primer to add a *Not* I site. Thus Jk1FOR derived primary PCR products are amplified by JK1FORNot as shown below, with the extension sequence in lower case and the *Not*I site underlined:

JK1FOR ACGTTTGATTTCCACCTTGGTCCC

JK1FORNot gagtcattctcgacttgcggccgcACGTTTGATTTCCACCTTGGTCCC

The VL primers sets with restriction sites added are shown below with the added restriction site underlined.

**Table S3. Sequence of primers used in secondary PCR of light chain variable region**

| VkBAKNheA | TCTGGCGGTGGCGCTAGCGACATCCAGATGACCCAG |
| --- | --- |
| VkBAKNheB | TCTGGCGGTGGCGCTAGCGATATTGTGATGACNCAG |
| VkBAKNheC | TCTGGCGGTGGCGCTAGCGAAACGACACTCACGCAG |
| VlBAKNheA | TCTGGCGGTGGCGCTAGCCAGTCTGNGCTGACTCAG |
| VlBAKNheB | TCTGGCGGTGGCGCTAGCTCCTATGAGCTGACTCAG |
| VlBAKNheC | TCTGGCGGTGGCGCTAGCAATTTTATGCTGACTCAG |
| JK1FORNot | GAGTCATTCTCGACTTGCGGCCGCACGTTTGATTTCCACCTTGGTCCC |
| JK2FORNot | GAGTCATTCTCGACTTGCGGCCGCACGTTTGATCTCCAGCTTGGTCCC |
| JK3FORNot | GAGTCATTCTCGACTTGCGGCCGCACGTTTGATATCCACTTTGGTCCC |
| JK4FORNot | GAGTCATTCTCGACTTGCGGCCGCACGTTTGATCTCCACCTTGGTCCC |
| JK5FORNot | GAGTCATTCTCGACTTGCGGCCGCACGTTTAATCTCCAGTCGTGTCCC |
| JL1FORNot | GAGTCATTCTCGACTTGCGGCCGCACCTAGGACGGTGACCTTGGTCCC |
| JL2-3FORNot | GAGTCATTCTCGACTTGCGGCCGCACCTAGGACGGTCAGCTTGGTCCC |
| JL4-5FORNot | GAGTCATTCTCGACTTGCGGCCGCACCTAAAACGGTGAGCTGGGTCCC |

Primary PCR product were generated using Hot Start Taq (Qiagen) with 40ng of template in a 50μl volume and Cycled 30 times at 95ºC- 1 min, 50-55ºC- 1 min; 72ºC- 1 min. Primary products were gel purified and 1/10^th^ of the product used as templates for secondary PCR to introduce restriction sites for cloning. Secondary PCR products were purified on PCR purification columns (Qiagen). At this stage products from the eleven cDNA template pools and the individual heavy, kappa and lambda chain families were all pooled together to create 7 heavy chain family pools, 6 kappa and 10 lambda pools reducing the sample number from 462 to 23. Products were digested with the appropriate restriction enzymes (New England Biolabs); gel purified and prepared using “Gel extraction kit” (Qiagen).

**Cloning of VL and VH repertoires into pSANG4.**

**Overview**. The VL repertoire was cloned as 9 separate sub-libraries (6 kappa families and 3 pools of lambda families) into an intermediate vector pSANG2 via the *Nhe*I/*Not*I sites to give an average of 1.1 10^7^ clones per library (10^8^ total). Plasmid DNA was prepared from this, pooled into kappa or lambda sets and the VH repertoire sub-cloned via *Nco*I/*Xho*I sites into these 2 light chain libraries as 7 separate sub-populations according to VH family to give a total of 14sub-libraries. In this intermediate vector, the VH genes were out of frame with the downstream elements and so the library was reconstructed from this intermediate library (3 x 10^10^ clones total) into pSANG4 by sequential cloning of VLs (again using *Nhe*I/*Not*I) and VHs (again using *Nco*I/*Xho*I) to give a final in frame library of 1.1 x 10^10^ clones.

**Initial VL cloning**. 2μg of *Nhe*I/*Not*I digested/SAP treated vector and 500ng digested insert were ligated using a Rapid DNA ligation kit (Roche) A total of 6 ligations representing each of the kappa gene families and 3 ligations representing 3 pools of the 10 lambda families (pool 1=Vλ 1, 2 and 3, pool 2= Vλ 4, 5 and 6, pool 3 =Vλ 7, 8, 4/9 and 10) were done. The ligations were purified using PCR purification columns (Qiagen) and eluted in 30μl water. The product was electroporated into fresh electrocompetent cells (efficiencies of 1 x 10^10^-1 x 10^11^) and cells plated onto plates with ampicillin and 2% glucose. 9 light chain sub-libraries were created with an average of 10^7^ clones/library. Sequencing confirmed the diversity of light chains in all sub-aliquots (not shown). Plasmid DNA was prepared from each of the library sub-aliquots, digested with *Nhe*I and *Not*I and pooled into a VL kappa set and a VL lambda set.

**Initial VH cloning.** Each of the 7 heavy chain families was used for separate ligations with either the kappa and lambda libraries, creating initially 14 separate libraries. Large scale ligations were performed at 16ºC overnight using T4 ligase (Roche) with 10μg of vector and 2μg of insert in 500μl. Ligations were purified using phenol:chloroform, ethanol precipitated and resuspended in water before electroporation. A total of 3 x 10^10^ clones were generated across 16 sub-aliquots.

**Re-cloning into pSANG4.** The cloned heavy and light chains described above were excised by their appropriate restriction enzymes and were re-cloned into pSANG4 which restores the correct frame of the heavy chain with the downstream elements. 9 different VL inserts, represented by kappa families 1-6 and lambda family pools 1-3(described above) were digested with *Nhe*I and *Not*I, gel purified and ligated with *Nhe*I/*Not*I cleaved pSANG4, essentially as described above. The total of the light chain library was 3x10^8^ with all showing 100% insert apart from lambda 1 with 9/10 with insert, as judged by PCR screening (not shown).

Vector DNA was prepared from the above light chain libraries, and digested with *Nco*I/*Xho*I. Vectors were treated with Antarctic phosphatase and then purified using Chromaspin-1000 DEPC columns (BD Biosciences). All intermediate libraries generated by kappa light chain cloning were pooled at this point as were the lambda libraries.

Heavy chain inserts were prepared from plasmid DNA of the previous libraries by *Nco*I/*Xho*I digestion followed by gel purification. Large scale ligations were set up and purified as before using 4μg of vector and 1μg of insert. 14 different sub-libraries were prepared, consisting of each of the 7 heavy chain groups combined with either kappa or lambda light chains intermediate libraries. Ewert *et al* (2003)^13^ suggest that particular combinations of VH and VL families express better and so 2 additional sub libraries of VH3/ κ3 and VH3/ κ1 were constructed to benefit from this. After electroporation, plating out and overnight growth, plates were scraped into freezing media (2xTY/15% glycerol/2% glucose) and aliquots were stored at -70°C. Number of colonies arising for each sub-library are shown below along with the proportion with insert, as judged by PCR screening. Taking into account the insert ratio from PCR screening, the total size of library was 1.1x10^10^.

**Table S4. Composition of the “McCafferty” scFv display library.**

| **Aliquot** | **Library size** | **Insertion ratio (colony PCR)** |
| --- | --- | --- |
| VH1κpSANG | 1.4x10^9^ | 100% |
| VH2κpSANG | 1.4x10^9^ | 82% |
| VH3κpSANG | 1.4x10^9^ | 91% |
| VH4κpSANG | 9.2x10^8^ | 91% |
| VH5κpSANG | 1.1x10^9^ | 91% |
| VH6κpSANG | 1.5x10^9^ | 91% |
| VH7κpSANG | 9.1x10^8^ | 91% |
| VH1λpSANG | 5.4x10^8^ | 100% |
| VH2λpSANG | 3.2x10^8^ | 82% |
| VH3λpSANG | 4.1x10^8^ | 100% |
| VH3λpSANG | 1.3x10^8^ | 100% |
| VH3λpSANG | 6.2x10^8^ | 91% |
| VH4λpSANG | 3.7x10^7^ | 73% |
| VH5λpSANG | 3.6x10^8^ | 73% |
| VH6λpSANG | 3.6x10^8^ | 73% |
| VH7λpSANG | 7.0x10^7^ | 73% |
| VH3κ1pSANG | 1.1x10^8^ | 73% |
| VH3κ1pSANG | 4.4x10^8^ | 91% |
| VH3κ3pSANG | 2.0x10^8^ | 82% |
| VH3κ3pSANG | 6.6x10^8^ | 82% |
| **TOTAL** | **1.3x10^10^**  **colonies** | **1.1 x 10^10^**  **with insert** |

**Production and QC of the McCafferty library for selection.**

**Phage preparation.** Each library aliquot was seeded at an optical density (OD_600nm_) of 0.1 (equivalent to 8x10^7^ cells/ml) in 500 ml of 2xTY/2% (w/v) glucose supplemented with ampicillin at a final concentration of 100 µg/ml in 2 L flasks. The bacteria were then incubated at 37°C, shaking until at mid-log phase (OD_600_ = 0.5). KM13 helper phage was added at a multiplicity of infection (MOI) of 10 and the cells incubated at 37°C for a further 30 min. without shaking, followed by 30 min. shaking slowly. The bacteria were then pelleted for 10 min. at 3000 rpm in 500 ml conical bottom centrifuge tubes. The supernatant was drained from the pellet, and the pellet resuspended in 500 ml 2xTY supplemented with both kanamycin (50 µg/ml) and ampicillin (100 µg/ml) in the absence of glucose. The bacterial cultures were then incubated overnight (16 h) at 25°C shaking.

**PEG precipitation of the phage library.** The 500 ml overnight cultures were centrifuged at 6000 rpm for 15 min. at 4 ºC. The supernatant was then decanted into a fresh 2 L flask and a 1/5^th^ volume of PEG solution [20% (w/v) polyethylene glycol (MW 8000), 2.5 M NaCl (Sigma)] added and mixed, before precipitating on ice for 1 h. The precipitated phage particles were pelleted by centrifugation at 6000 rpm for 15 min. at 4ºC. The supernatant was discarded, and the pellet resuspended in 12 ml T0.1E solution [1.0 M Tris HCl (pH 8.0), 0.1 M EDTA]. The resuspended pellet was then centrifuged at 10,000 x **g** at 4ºC for 30 min. to remove any remaining bacterial cells and debris. The supernatant was transferred into a fresh tube, and 0.5 g of cesium chloride added for each 1 ml of supernatant. After thorough mixing by inversion, the tubes were incubated on ice for 10 min. NaN_3_ was added to a final concentration of 0.02% (w/v). The phage preparation was then loaded into 39 ml ultracentrifuge tubes (Beckman) and topped up with T0.1E/CsCl [0.5 g cesium chloride per 1 ml T0.1E solution]. The tubes were then centrifuged at 40,000 rpm for 60 h. at 20°C in a 70 Ti rotor (Beckman). The phage band was removed from the tube using a 20Gx1½” needle and 1 ml syringe, added to a fresh ultracentrifuge tube, diluted again in T0.1E/CsCl and centrifuged at 40,000 rpm for 60 h. at 20°C. Again the band was carefully removed using a needle and syringe and the recovered phage particles were dialyzed overnight at 4°C against TE solution [10mM Tris HCl (pH 8.0), 1mM EDTA]. The next day after dialysis, NaN_3_ was added to a final concentration of 0.02% (w/v).

**Titrating the library.** Cells infected with the antibody-bearing phage particles become resistant to ampicillin. The titer of the phage can be determined simply by plating out TG1 cells infected with dilutions of the cesium chloride purified phage onto 2xTY/glu/amp plates. By counting the number of colonies on these dishes, the titer of phage in the original culture can be determined. The expected titer of a given phage preparation is between 10^12^ to 10^14^ phage per ml. It is important to plate out 100 µl of uninfected TG1 cells to ensure that the host cells are not already infected. One ml of TG1 cells (OD_600_ = 1.0 (8x10^8^ cells/ml)) was added to each well of a 2 ml deep well plate, then 100 µl of each log_10_ dilution of phage preparation was added to the cells, and incubated shaking slowly for 1 h at 37°C. The cells were then pelleted at 3000 rpm for 5 min., the supernatant discarded and the cell pellet resuspended in 100 µl 2xTY media. The cells were then spread onto 2xTY/glu/amp plates and incubated at 37°C overnight. The next day the colonies were counted and the phage preparation titer determined.

**QC of the library by western blot.** The quality control process for the library was performed monthly on example aliquots of each of the phage library preparations. We used a library preparation supplied by Cambridge Antibody Technology as a control sample. For each aliquot we loaded 3.5x10^11^ phage particles per lane into an SDS-PAGE gel and ran the samples under reducing and denaturing conditions. The separated phage proteins were then transferred to a PVDF membrane for western blot analysis. The membrane was blocked in 3% skimmed milk (w/v) PBS with 0.1% (v/v) Tween-20 (M-PBS-T) for 1 h at room temperature. The membrane was then probed with a 1:2000 dilution of anti-M13 pIII (New England BioLabs) diluted in M-PBS-T for 1 h at room temperature. The membrane was then washed three times in PBS-T, prior to the addition of a 1:2500 dilution of anti mouse IgG–Cy5 (Amersham Biosciences) and incubated in the dark for 1 h at room temperature. Following washing, as above, the membrane was rinsed once in deionized water and dried in the dark before being scanned on a Typhoon 9410 scanner (Amersham Biosciences) and analyzed using the ImageQuant software package (Amersham Biosciences).

**References**

3. Marks, J.D. et al. By-passing immunization. Human antibodies from V-gene libraries displayed on phage. *J Mol Biol* **222**, 581-597 (1991).

4. Ewert, S., Honegger, A. & Pluckthun, A. Structure-based improvement of the biophysical properties of immunoglobulin VH domains with a generalizable approach. *Biochemistry* **42**, 1517-1528 (2003).
