# Supplementary material for: Application of phage display to high throughput antibody generation and characterization
Source: Genome Biol. 2007 Nov 29;8(11):R254. doi: 10.1186/gb-2007-8-11-r254 (PMC2258204; doi:10.1186/gb-2007-8-11-r254)
Supplement: Additional data file 2 — Median fluorescent intensities of antigen beads as plotted in Figure 5. [file gb-2007-8-11-r254-S2.doc]

**Additional data file 2**

**Table 1. List of 406 antigens used in antibody selection and the number of unique antibodies generated.**

The gene symbol nomenclature used is according to MGI (http://www.informatics.jax.org/) and HUGO (http://www.gene.ucl.ac.uk/nomenclature/) for mouse and human genes respectively. “Antigen name” is the tracking ID used within the Atlas LIMS system. “Sequence uniques” refers to the number of sequence unique scFv clone identified.

| **Antigen name** | **Gene symbol** | **Antigen source** | **Expression_host** | **Sequence uniques** |
| --- | --- | --- | --- | --- |
| ant1 | EPHA1 | R&D Systems | Mammalian | 30 |
| ant2 | Epha4 | R&D Systems | Mammalian | 31 |
| ant3 | Epha2 | R&D Systems | Mammalian | 22 |
| ant4 | Epha3 | R&D Systems | Mammalian | 32 |
| ant5 | Epha5 | R&D Systems | Mammalian | 30 |
| ant6 | Epha7 | R&D Systems | Mammalian | 24 |
| ant7 | Epha8 | R&D Systems | Mammalian | 16 |
| ant10 | Ephb3 | R&D Systems | Mammalian | 44 |
| ant11 | Ephb4 | R&D Systems | Mammalian | 32 |
| ant12 | Ephb6 | R&D Systems | Mammalian | 35 |
| ant13 | Efna1 | R&D Systems | Mammalian | 44 |
| ant14 | Efna2 | R&D Systems | Mammalian | 37 |
| ant15 | EFNA3 | R&D Systems | Mammalian | 43 |
| ant16 | Efna4 | R&D Systems | Mammalian | 23 |
| ant17 | EFNA5 | R&D Systems | Mammalian | 19 |
| ant18 | Efnb1 | R&D Systems | Mammalian | 4 |
| ant19 | Efnb2 | R&D Systems | Mammalian | 41 |
| ant20 | EFNB3 | R&D Systems | Mammalian | 32 |
| ant21 | TNFRSF10C | R&D Systems | Mammalian | 10 |
| ant22 | Epha6 | R&D Systems | Mammalian | 36 |
| ant8 | Ephb1 | R&D Systems | Mammalian | 14 |
| ant9 | Ephb2 | R&D Systems | Mammalian | 41 |
| ant23 | Tnfrsf9 | R&D Systems | Mammalian | 56 |
| ant24 | Tnfrsf7 | R&D Systems | Mammalian | 16 |
| ant25 | Tnfrsf13c | R&D Systems | Mammalian | 26 |
| ant26 | Tnfrsf17 | R&D Systems | Mammalian | 58 |
| ant27 | Tnfrsf8 | R&D Systems | Mammalian | 29 |
| ant28 | Tnfrsf5 | R&D Systems | Mammalian | 35 |
| ant29 | TNFRSF25 | R&D Systems | Mammalian | 7 |
| ant30 | TNFRSF21 | R&D Systems | Insect | 52 |
| ant31 | Sstr4 | Atlas | Bacterial | 30 |
| ant31 | Sstr4 | Atlas | Bacterial | 0 |
| ant32 | Sstr2 | Atlas | Bacterial | 28 |
| ant32 | Sstr2 | Atlas | Bacterial | 0 |
| ant33 | Ly6g6d | Atlas | Bacterial | 11 |
| ant34 | Lama1 | Atlas | Bacterial | 53 |
| ant35 | ELF1 | Atlas | Bacterial | 13 |
| ant36 | Fli1 | Atlas | Bacterial | 22 |
| ant37 | Gatad2a | Atlas | Bacterial | 30 |
| ant41 | Myo7a | Atlas | Bacterial | 15 |
| ant42 | Fli1 | Atlas | Bacterial | 24 |
| ant483 | Snx15 | Atlas | Bacterial | 27 |
| ant484 | Pin1 | Atlas | Bacterial | 13 |
| ant485 | Snx15 | Atlas | Bacterial | 33 |
| **Antigen name** | **Gene symbol** | **Antigen source** | **Expression_host** | **Sequence uniques** |
| ant118 | Tnfrsf19 | R&D Systems | Insect | 41 |
| ant119 | Tnfrsf12a | R&D Systems | Insect | 29 |
| ant120 | EDA2R | R&D Systems | Mammalian | 51 |
| ant121 | Tnfrsf1a | R&D Systems | Mammalian | 29 |
| ant122 | Tnfrsf1b | R&D Systems | Bacterial | 58 |
| ant42 | Fli1 | Atlas | Bacterial | 18 |
| ant43 | Bsg | Atlas | Mammalian | 62 |
| ant44 | Elf1 | Atlas | Bacterial | 23 |
| ant45 | Gata2 | Atlas | Bacterial | 14 |
| ant46 | ELF1 | Atlas | Bacterial | 25 |
| ant47 | Gata1 | Atlas | Bacterial | 13 |
| ant48 | Gata1 | Atlas | Bacterial | 25 |
| ant49 | Fli1 | Atlas | Bacterial | 43 |
| ant50 | Edar | R&D Systems | Mammalian | 45 |
| ant51 | Fas | R&D Systems | Insect | 25 |
| ant52 | TNFRSF18 | R&D Systems | Mammalian | 51 |
| ant53 | TNFRSF14 | R&D Systems | Mammalian | 50 |
| ant54 | Ngfr | R&D Systems | Mammalian | 56 |
| ant55 | Tnfrsf11b | R&D Systems | Mammalian | 75 |
| ant56 | Tnfrsf4 | R&D Systems | Mammalian | 89 |
| ant57 | Tnfrsf11a | R&D Systems | Mammalian | 40 |
| ant58 | TNFRSF19L | R&D Systems | Mammalian | 65 |
| ant59 | Tnfrsf13b | R&D Systems | Mammalian | 31 |
| ant104 | Homer1 | Atlas | Bacterial | 0 |
| ant105 | Myo7a | Atlas | Bacterial | 18 |
| ant113 | Myo15 | Atlas | Bacterial | 0 |
| ant114 | TNFRSF10B | R&D Systems | Mammalian | 63 |
| ant125 | Tnfrsf22 | R&D Systems | Mammalian | 58 |
| ant127 | FLJ20085 | Atlas | Bacterial | 37 |
| ant135 | FLJ20085 | Atlas | Bacterial | 0 |
| ant60 | Espn | Atlas | Bacterial | 37 |
| ant62 | TNFRSF10D | R&D Systems | Mammalian | 23 |
| ant67 | Atp2b2 | Atlas | Bacterial | 0 |
| ant70 | Cdh23 | Atlas | Bacterial | 0 |
| ant74 | TNFRSF10A | R&D Systems | Mammalian | 60 |
| ant77 | Ldb1 | Atlas | Bacterial | 0 |
| ant84 | Whrn | Atlas | Bacterial | 0 |
| ant89 | Whsc1 | Atlas | Bacterial | 0 |
| ant95 | Fli1 | Atlas | Bacterial | 0 |
| ant96 | Whsc1 | Atlas | Bacterial | 1 |
| ant98 | Whsc1 | Atlas | Bacterial | 0 |
| ant99 | Mtap2 | Atlas | Bacterial | 2 |
| ant101 | TAL1 | Atlas | Bacterial | 0 |
| ant106 | Gata2 | Atlas | Bacterial | 4 |
| ant107 | Gata1 | Atlas | Bacterial | 0 |
| ant115 | OR1D2 | Atlas | Bacterial | 1 |
| ant133 | KIAA1136 | Atlas | Bacterial | 2 |
| ant66 | Lyl1 | Atlas | Bacterial | 1 |
| ant69 | OR1D2 | Atlas | Bacterial | 9 |
| ant72 | Whrn | Atlas | Bacterial | 13 |
| ant75 | C11orf41 | Atlas | Bacterial | 6 |
| ant76 | Myog | Atlas | Bacterial | 1 |
| ant78 | Elf1 | Atlas | Bacterial | 6 |
| **Antigen name** | **Gene symbol** | **Antigen source** | **Expression_host** | **Sequence uniques** |
| ant79 | Homer3 | Atlas | Bacterial | 0 |
| ant81 | Gata1 | Atlas | Bacterial | 5 |
| ant82 | Fli1 | Atlas | Bacterial | 3 |
| ant87 | Gata2 | Atlas | Bacterial | 13 |
| ant88 | Relb | Atlas | Bacterial | 0 |
| ant91 | Dlgh3 | Atlas | Bacterial | 2 |
| ant93 | Dlgh4 | Atlas | Bacterial | 0 |
| ant94 | Dlgh1 | Atlas | Bacterial | 0 |
| ant137 | 1500001H12 | Atlas | Bacterial | 0 |
| ant139 | Ttr | Atlas | Bacterial | 2 |
| ant141 | SynGAP | Atlas | Bacterial | 0 |
| ant143 | Psd93 or Dlgh2 | Atlas | Bacterial | 2 |
| ant145 | Psd93 or Dlgh2 | Atlas | Bacterial | 7 |
| ant147 | MGC19339 | Atlas | Bacterial | 7 |
| ant149 | KIAA1136 | Atlas | Bacterial | 0 |
| ant151 | KIAA1549 | Atlas | Bacterial | 0 |
| ant153 | Fli1 | Atlas | Bacterial | 3 |
| ant155 | Cd4 | Atlas | Mammalian | 17 |
| ant157 | LAIR1 | Atlas | Mammalian | 37 |
| ant159 | CD83 | Atlas | Mammalian | 29 |
| ant161 | Cd2 | Atlas | Mammalian | 55 |
| ant163 | CD5 | Atlas | Mammalian | 44 |
| ant165 | CD5 | Atlas | Mammalian | 47 |
| ant167 | CD47 | Atlas | Mammalian | 40 |
| ant169 | Cd200 | Atlas | Mammalian | 37 |
| ant171 | Efnb2 | Atlas | Mammalian | 51 |
| ant173 | C1QTNF5 | Collaborator | Bacterial | 56 |
| ant175 | C1QTNF5 | Atlas | Bacterial | 41 |
| ant177 | MFRP | Atlas | Bacterial | 0 |
| ant179 | MFRP | Atlas | Bacterial | 7 |
| ant296 | Jam4 | Atlas | Mammalian | 0 |
| ant38 | Snx15 | Atlas | Bacterial | 28 |
| ant39 | Pin1 | Atlas | Bacterial | 11 |
| ant40 | Snx15 | Atlas | Bacterial | 32 |
| ant493 | Homer1 | Atlas | Bacterial | 0 |
| ant93 | Dlgh4 | Atlas | Bacterial | 0 |
| ant280 | Fli1 | Atlas | Bacterial | 0 |
| ant282 | Fli1 | Atlas | Bacterial | 0 |
| ant284 | Fli1 | Atlas | Bacterial | 0 |
| ant288 | Nfkb1 | Atlas | Bacterial | 1 |
| ant290 | RelA-p65 | Atlas | Bacterial | 3 |
| ant292 | ELF1 | Atlas | Bacterial | 0 |
| ant294 | Elf1 | Atlas | Bacterial | 0 |
| ant296 | Jam4 | Atlas | Mammalian | 0 |
| ant298 | Efnb2 | Atlas | Mammalian | 12 |
| ant300 | CDH2 | R&D Systems | Mammalian | 12 |
| ant302 | CDH3 | R&D Systems | Mammalian | 5 |
| ant304 | CDH1 | R&D Systems | Mammalian | 3 |
| ant306 | Flt1 | R&D Systems | Mammalian | 6 |
| ant71 | Mtap2 | Atlas | Bacterial | 0 |
| ant308 | Rab9B | Atlas | Bacterial | 38 |
| ant310 | Rab32 | Atlas | Bacterial | 56 |
| ant312 | Rab9A | Atlas | Bacterial | 37 |
| **Antigen name** | **Gene symbol** | **Antigen source** | **Expression_host** | **Sequence uniques** |
| ant314 | Rab6A-2 | Atlas | Bacterial | 20 |
| ant316 | Rab44 | Atlas | Bacterial | 41 |
| ant318 | Rab6A-1 | Atlas | Bacterial | 21 |
| ant320 | Rab7 | Atlas | Bacterial | 46 |
| ant322 | Rab17 | Atlas | Bacterial | 22 |
| ant324 | Rab38 | Atlas | Bacterial | 35 |
| ant326 | Rab6B | Atlas | Bacterial | 30 |
| ant328 | Rab28 | Atlas | Bacterial | 2 |
| ant330 | Rab29 | Atlas | Bacterial | 24 |
| ant345 | Cd226 | Atlas | Mammalian | 11 |
| ant347 | Gp49a | Atlas | Mammalian | 0 |
| ant349 | Il18rap | Atlas | Mammalian | 0 |
| ant350 | Slamf7 | Atlas | Mammalian | 33 |
| ant352 | Cd2 | Atlas | Mammalian | 0 |
| ant353 | Mfap3l | Atlas | Mammalian | 0 |
| ant356 | Ptpns1 | Atlas | Mammalian | 0 |
| ant357 | Cd86 | Atlas | Mammalian | 23 |
| ant361 | Icam2 | Atlas | Mammalian | 40 |
| ant362 | Esam1 | Atlas | Mammalian | 44 |
| ant363 | Gp49a | Atlas | Mammalian | 0 |
| ant364 | Esam1 | Atlas | Mammalian | 28 |
| ant365 | Il6ra | Atlas | Mammalian | 52 |
| ant366 | Il1rap | Atlas | Mammalian | 22 |
| ant367 | Cd86 | Atlas | Mammalian | 34 |
| ant368 | D330012D11Rik | Atlas | Mammalian | 0 |
| ant371 | Vsig4 | Atlas | Mammalian | 56 |
| ant372 | Fcrl1 | Atlas | Mammalian | 40 |
| ant373 | Vsig1 | Atlas | Mammalian | 55 |
| ant374 | Vsig1 | Atlas | Mammalian | 48 |
| ant160 | Ptpns1 | Atlas | Mammalian | 49 |
| ant344 | Mpz | Atlas | Mammalian | 0 |
| ant346 | 2200002k05Rik | Atlas | Mammalian | 0 |
| ant348 | Cd226 | Atlas | Mammalian | 16 |
| ant351 | Ildr1 | Atlas | Mammalian | 48 |
| ant354 | Cd7 | Atlas | Mammalian | 19 |
| ant355 | Pdcd1 | Atlas | Mammalian | 0 |
| ant358 | Pigr | Atlas | Mammalian | 4 |
| ant359 | Slamf6 | Atlas | Mammalian | 5 |
| ant360 | Axl | Atlas | Mammalian | 51 |
| ant369 | Mpz | Atlas | Mammalian | 22 |
| ant370 | 4632428N05Rik | Atlas | Mammalian | 0 |
| ant190 | Ctla4 | Atlas | Bacterial | 0 |
| ant205 | B430306N03Rik | Atlas | Bacterial | 0 |
| ant208 | B430306N03Rik | Atlas | Bacterial | 0 |
| ant210 | Sectm1 | Atlas | Bacterial | 0 |
| ant212 | Timd2 | Atlas | Bacterial | 1 |
| ant213 | Treml1 | Atlas | Bacterial | 0 |
| ant223 | Cd200r1 | Atlas | Bacterial | 0 |
| ant227 | Lilrb4 | Atlas | Bacterial | 0 |
| ant241 | 2200002K05Rik | Atlas | Bacterial | 3 |
| ant255 | Jam4 | Atlas | Bacterial | 0 |
| ant273 | 4632428N05Rik | Atlas | Bacterial | 0 |
| ant430 | Fcgr2b | Atlas | Bacterial | 0 |
| **Antigen name** | **Gene symbol** | **Antigen source** | **Expression_host** | **Sequence uniques** |
| ant431 | B430306N03Rik | Atlas | Bacterial | 0 |
| ant433 | Eva1 | Atlas | Bacterial | 0 |
| ant434 | Cd300lf | Atlas | Bacterial | 1 |
| ant435 | 1700001D09Rik | Atlas | Bacterial | 0 |
| ant437 | Cd300lf | Atlas | Bacterial | 0 |
| ant438 | Fcer1a | Atlas | Bacterial | 0 |
| ant441 | Treml1 | Atlas | Bacterial | 0 |
| ant442 | F11r | Atlas | Bacterial | 8 |
| ant443 | Eva1 | Atlas | Bacterial | 0 |
| ant444 | F11r | Atlas | Bacterial | 30 |
| ant445 | Lilrb4 | Atlas | Bacterial | 0 |
| ant446 | B430306N03Rik | Atlas | Bacterial | 0 |
| ant448 | Pilra | Atlas | Bacterial | 1 |
| ant450 | Cd7 | Atlas | Bacterial | 0 |
| ant451 | Fcer1a | Atlas | Bacterial | 0 |
| ant452 | Havcr2 | Atlas | Bacterial | 0 |
| ant453 | Cd7 | Atlas | Bacterial | 0 |
| ant454 | Scn3b | Atlas | Bacterial | 0 |
| ant455 | Pdcd1 | Atlas | Bacterial | 4 |
| ant457 | Ctla4 | Atlas | Bacterial | 0 |
| ant458 | Mpz | Atlas | Bacterial | 0 |
| ant459 | Cd84 | Atlas | Bacterial | 0 |
| ant464 | Lilrb4 | Atlas | Bacterial | 0 |
| ant465 | Jam4 | Atlas | Bacterial | 13 |
| ant466 | 1700001D09Rik | Atlas | Bacterial | 0 |
| ant467 | Pvrl4 | Atlas | Bacterial | 9 |
| ant470 | Cd79b | Atlas | Bacterial | 0 |
| ant471 | Pdcd1 | Atlas | Bacterial | 4 |
| ant476 | 2200002K05Rik | Atlas | Bacterial | 2 |
| ant477 | Fcrl5 | Atlas | Bacterial | 0 |
| ant478 | Mpz | Atlas | Bacterial | 0 |
| ant480 | Cd300lg | Atlas | Bacterial | 0 |
| ant481 | Scn3b | Atlas | Bacterial | 0 |
| ant482 | 4632428N05Rik | Atlas | Bacterial | 4 |
| ant188 | Ager | Atlas | Bacterial | 1 |
| ant195 | Kit | Atlas | Bacterial | 1 |
| ant202 | Cd4 | Atlas | Bacterial | 0 |
| ant226 | Lrrn2 | Atlas | Bacterial | 0 |
| ant230 | Igsf4b | Atlas | Bacterial | 0 |
| ant231 | Timd2 | Atlas | Bacterial | 0 |
| ant235 | Kit | Atlas | Bacterial | 0 |
| ant238 | Pecam1 | Atlas | Bacterial | 0 |
| ant245 | Cd84 | Atlas | Bacterial | 0 |
| ant249 | Gp49a | Atlas | Bacterial | 0 |
| ant251 | Lrrn1 | Atlas | Bacterial | 1 |
| ant256 | Siglec10 | Atlas | Bacterial | 0 |
| ant266 | Fcrl1 | Atlas | Bacterial | 14 |
| ant429 | Fcgrt | Atlas | Bacterial | 7 |
| ant439 | Cd86 | Atlas | Bacterial | 20 |
| ant456 | Lrrn1 | Atlas | Bacterial | 0 |
| ant460 | Lrrc14 | Atlas | Bacterial | 0 |
| ant461 | F11r | Atlas | Bacterial | 5 |
| ant469 | Lilrb4 | Atlas | Bacterial | 0 |
| **Antigen name** | **Gene symbol** | **Antigen source** | **Expression_host** | **Sequence uniques** |
| ant472 | Cd86 | Atlas | Bacterial | 6 |
| ant473 | Ager | Atlas | Bacterial | 0 |
| ant474 | Kit | Atlas | Bacterial | 0 |
| ant475 | Cd226 | Atlas | Bacterial | 0 |
| ant487 | twist1 | Atlas | Bacterial | 8 |
| ant488 | MLR10 | Atlas | Bacterial | 15 |
| ant489 | MLR10 | Atlas | Bacterial | 1 |
| ant490 | MLR10 | Atlas | Bacterial | 1 |
| ant497 | Ceacam2 | Atlas | Mammalian | 3 |
| ant498 | Lrrn6c | Atlas | Mammalian | 20 |
| ant499 | Havcr2 | Atlas | Mammalian | 30 |
| ant500 | 6430556C10Rik | Atlas | Mammalian | 0 |
| ant501 | Lrrn6a | Atlas | Mammalian | 0 |
| ant502 | Pilra | Atlas | Mammalian | 23 |
| ant503 | Igsf4b | Atlas | Mammalian | 3 |
| ant504 | Cd79b | Atlas | Mammalian | 0 |
| ant505 | Tcam1 | Atlas | Mammalian | 0 |
| ant506 | Cd79b | Atlas | Mammalian | 1 |
| ant507 | Fcgr3a | Atlas | Mammalian | 36 |
| ant508 | Ager | Atlas | Mammalian | 0 |
| ant509 | Ctla4 | Atlas | Mammalian | 0 |
| ant510 | Mag | Atlas | Mammalian | 27 |
| ant511 | Slamf9 | Atlas | Mammalian | 30 |
| ant513 | Ceacam2 | Atlas | Mammalian | 31 |
| ant177 | MFRP | Atlas | Bacterial | 0 |
| ant25 | Tnfrsf13c | R&D Systems | Mammalian | 1 |
| ant26 | Tnfrsf17 | R&D Systems | Mammalian | 24 |
| ant300 | CDH2 | R&D Systems | Mammalian | 26 |
| ant302 | CDH3 | R&D Systems | Mammalian | 9 |
| ant304 | CDH1 | R&D Systems | Mammalian | 4 |
| ant480 | Cd300lg | Atlas | Bacterial | 36 |
| ant526 | Timd4 | Atlas | Mammalian | 12 |
| ant527 | Cd8a | Atlas | Mammalian | 11 |
| ant528 | Emb | Atlas | Mammalian | 2 |
| ant529 | Ly9 | Atlas | Mammalian | 5 |
| ant530 | undefined | Atlas | Mammalian | 0 |
| ant531 | Cd47 | Atlas | Mammalian | 9 |
| ant532 | Btla | Atlas | Mammalian | 13 |
| ant533 | Il1r2 | Atlas | Mammalian | 4 |
| ant534 | Cd274 | Atlas | Mammalian | 0 |
| ant535 | D7Ertd458e | Atlas | Mammalian | 28 |
| ant536 | Pecam1 | Atlas | Mammalian | 25 |
| ant537 | Fcer1a | Atlas | Mammalian | 29 |
| ant538 | 9030425E11Rik | Atlas | Mammalian | 0 |
| ant539 | Trem1 | Atlas | Mammalian | 37 |
| ant540 | Pdcd1lg2 | Atlas | Mammalian | 8 |
| ant541 | Lag3 | Atlas | Mammalian | 0 |
| ant542 | Unc5b | Atlas | Mammalian | 34 |
| ant543 | NP_766211 | Atlas | Mammalian | 3 |
| ant544 | Cd200 | Atlas | Mammalian | 6 |
| ant545 | Gpa33 | Atlas | Mammalian | 31 |
| ant546 | A530064D06Rik | Atlas | Mammalian | 8 |
| ant547 | Cd300d | Atlas | Mammalian | 0 |
| **Antigen name** | **Gene symbol** | **Antigen source** | **Expression_host** | **Sequence uniques** |
| ant548 | Cd2 | Atlas | Mammalian | 5 |
| ant549 | C130076O07Rik | Atlas | Mammalian | 46 |
| ant551 | Cd200r1 | Atlas | Mammalian | 0 |
| ant552 | Plaur | R&D Systems | Mammalian | 29 |
| ant553 | Collagen Type III | US Biologicals | Mammalian | 75 |
| ant554 | Collagen Type IV | US Biologicals | Mammalian | 29 |
| ant555 | TFRC | Biogenesis | Mammalian | 36 |
| ant556 | Hspb1 | US Biologicals | Bacterial | 14 |
| ant557 | HSP90AA1 | US Biologicals | Bacterial | 10 |
| ant558 | CD40 | ABNOVA | In vitro (wheat germ) | 2 |
| ant559 | FCER1A | ABNOVA | In vitro (wheat germ) | 3 |
| ant560 | AGER | ABNOVA | In vitro (wheat germ) | 5 |
| ant561 | EPHA4 | ABNOVA | In vitro (wheat germ) | 10 |
| ant562 | EDA2R | ABNOVA | In vitro (wheat germ) | 5 |
| ant529 | Ly9 | Atlas | Mammalian | 15 |
| ant530 | undefined | Atlas | Mammalian | 8 |
| ant540 | Pdcd1lg2 | Atlas | Mammalian | 26 |
| ant548 | Cd2 | Atlas | Mammalian | 19 |
| ant563 | Nope | R&D Systems | Mammalian | 38 |
| ant564 | Jam3 | R&D Systems | Mammalian | 48 |
| ant565 | Icam5 | R&D Systems | Mammalian | 66 |
| ant566 | F11r | R&D Systems | Mammalian | 22 |
| ant567 | Alcam | R&D Systems | Mammalian | 31 |
| ant568 | Il13ra1 | R&D Systems | Mammalian | 19 |
| ant569 | Cd86 | R&D Systems | Insect | 45 |
| ant570 | Cd80 | R&D Systems | Mammalian | 47 |
| ant571 | Pdcd1 | R&D Systems | Mammalian | 0 |
| ant572 | Jam2 | R&D Systems | Mammalian | 11 |
| ant573 | Pigr | R&D Systems | Mammalian | 48 |
| ant574 | Fcgr2b | R&D Systems | Mammalian | 21 |
| ant575 | Sigirr | R&D Systems | Mammalian | 37 |
| ant576 | Tek | R&D Systems | Mammalian | 24 |
| ant577 | Il1rl2 | R&D Systems | Mammalian | 9 |
| ant578 | Cd14 | R&D Systems | Mammalian | 10 |
| ant579 | Fasl | R&D Systems | Mammalian | 20 |
| ant580 | Tnfsf7 | R&D Systems | Mammalian | 41 |
| ant581 | Mpl | R&D Systems | Mammalian | 44 |
| ant582 | Cd22 | R&D Systems | Mammalian | 46 |
| ant583 | Siglecf | R&D Systems | Mammalian | 29 |
| ant584 | Fcgr1 | R&D Systems | Mammalian | 6 |
| ant585 | Cd36 | R&D Systems | Mammalian | 29 |
| ant586 | Il1r1 | R&D Systems | Mammalian | 51 |
| ant587 | Pdgfra | R&D Systems | Mammalian | 31 |
| ant588 | Vcam1 | R&D Systems | Mammalian | 41 |
| ant589 | Dcc | R&D Systems | Insect | 56 |
| ant590 | Il2ra | R&D Systems | Mammalian | 31 |
| ant591 | Cd40lg | R&D Systems | Mammalian | 0 |
| ant592 | Pdgfrb | R&D Systems | Mammalian | 36 |
| ant593 | Cd83 | R&D Systems | Insect | 24 |
| **Antigen name** | **Gene symbol** | **Antigen source** | **Expression_host** | **Sequence uniques** |
| ant594 | Trem2 | R&D Systems | Mammalian | 9 |
| ant595 | Madcam1 | R&D Systems | Mammalian | 2 |
| ant596 | Eng | R&D Systems | Mammalian | 35 |
| ant597 | F3 | Collaborator | Bacterial | 5 |
| ant659 | Il17rb | Atlas | Mammalian | 6 |
| ant660 | NP_573472 | Atlas | Mammalian | 40 |
| ant661 | Lrrn1 | Atlas | Mammalian | 7 |
| ant662 | Tmem27 | Atlas | Mammalian | 5 |
| ant663 | Gpa33 | Atlas | Mammalian | 20 |
| ant664 | AI415330 | Atlas | Mammalian | 2 |
| ant665 | 4930572D21Rik | Atlas | Mammalian | 0 |
| ant666 | Igl-V1 | Atlas | Mammalian | 35 |
| ant667 | Robo2 | Atlas | Mammalian | 28 |
| ant668 | Sema7a | Atlas | Mammalian | 40 |
| ant669 | undefined | Atlas | Mammalian | 4 |
| ant670 | 2010106E10Rik | Atlas | Mammalian | 15 |
| ant671 | 6330527O06Rik | Atlas | Mammalian | 5 |
| ant672 | Pvrl2 | Atlas | Mammalian | 17 |
| ant673 | 0610031J06Rik | Atlas | Mammalian | 8 |
| ant674 | Il1rl1 | Atlas | Mammalian | 2 |
| ant675 | Crry | Atlas | Mammalian | 31 |
| ant676 | Lamp2 | Atlas | Mammalian | 27 |
| ant677 | Bambi | Atlas | Mammalian | 7 |
| ant678 | Fas | Atlas | Mammalian | 21 |
| ant679 | Cd19 | Atlas | Mammalian | 17 |
| ant680 | Pdpn | Atlas | Mammalian | 12 |
| ant681 | Tnfrsf19 | Atlas | Mammalian | 1 |
| ant682 | Cd68 | Atlas | Mammalian | 3 |
| ant683 | Zp3 | Atlas | Mammalian | 35 |
| ant684 | 1500004A08Rik | Atlas | Mammalian | 6 |
| ant685 | Plat | Atlas | Mammalian | 6 |
| ant686 | Mansc1 | Atlas | Mammalian | 17 |
| ant687 | Havcr1 | Atlas | Mammalian | 29 |
| ant688 | GPVI | Collaborator | Bacterial | 17 |
| ant689 | Gpvi | Collaborator | Bacterial | 42 |
| ant690 | MT1-MMP | Collaborator | Bacterial | 25 |
| ant691 | MT2-MMP | Collaborator | Bacterial | 24 |
| ant692 | MT3-MMP | Collaborator | Bacterial | 38 |
| ant693 | Peptide A | Collaborator |  | 49 |
| ant694 | Peptide B | Collaborator |  | 41 |
| ant695 | Peptide C | Collaborator |  | 59 |
| ant696 | Peptide D | Collaborator |  | 66 |
| ant697 | Peptide E | Collaborator |  | 53 |
| ant698 | Peptide F | Collaborator |  | 54 |
| ant699 | Pecam1 D11 | Atlas | Bacterial | 36 |
| ant700 | CTLA4 | Atlas | Bacterial | 0 |
| ant701 | CTLA4 | Atlas | Bacterial | 0 |
| ant702 | CTLA4 | Atlas | Bacterial | 0 |
