# Supplementary material for: Application of phage display to high throughput antibody generation and characterization
Source: Genome Biol. 2007 Nov 29;8(11):R254. doi: 10.1186/gb-2007-8-11-r254 (PMC2258204; doi:10.1186/gb-2007-8-11-r254)
Supplement: Additional data file 3 — Description of staining profiles supported by multiple antibodies in immunohistochemistry. [file gb-2007-8-11-r254-S3.doc]

**Additional data file 2**

**Supplementary Table 2. Median fluorescent intensities of antigen beads as plotted in figure 5 Schofield et al, 2007**

Flow cytometry calibration beads were coated with various antigen densities/bead, analysed by flow cytometry and the relative median fluorescent intensity of the bead with 459,000 copies/bead was calculated (figure 5 of Schofield et al, 2007). Below are the actual values and the clone identifications associated with them. At the flow cytometry settings used, the uncoated bead gave a score of 0.1-0.2 and figure 5 plots the ratio of median fluorescent intensity for the coated and uncoated bead “UN” means signal from bead with 459,000 copies/bead was unresolved from lower density beads i.e. a low intensity signal.

| Efna2 | | Efna4 | | Plaur | | Alcam | |
| --- | --- | --- | --- | --- | --- | --- | --- |
| **Antibody** | **Score** | **Antibody** | **Score** | **Antibody** | **Score** | **Antibody** | **Score** |
| ant14_19 - D10 | 182.0 | ant16_21 - D02 | 402 | ant552_360 - D03 | 165 | ant567_733 -C10 | 296 |
| ant14_19 - E12 | 162.0 | ant16_21 - E09 | 384 | ant552_360 - D01 | 117 | ant567_733 - F10 | 271 |
| ant14_19 - C03 | 151.0 | ant16_21 - G10 | 361 | ant552_360 - B12 | 106 | ant567_733 -C01 | 216 |
| ant14_19 - G10 | 84.8 | ant16_21 - B03 | 357 | ant552_360 - F08 | 54.1 | ant567_733 - F11 | 205 |
| ant14_19 - D09 | 84.0 | ant16_21 - A06 | 271 | ant552_360 - C12 | 44 | ant567_733 -A02 | 194 |
| ant14_19 - H11 | 70.8 | ant16_21 - E02 | 254 | ant552_360 - A07 | 39.4 | ant567_733 -G01 | 149 |
| ant14_19 - B09 | 60.8 | ant16_21 - G01 | 232 | ant552_360 - H04 | 36.1 | ant567_733 -B08 | 111 |
| ant14_19 - A11 | 14.9 | ant16_21 - B01 | 176 | ant552_360 - B06 | UN | ant567_733 - F01 | 101 |
| ant14_19 - G06 | 2.3 | ant16_21 - A02 | 125 | ant552_360 - A05 | UN | ant567_733 - F09 | 85.5 |
| ant14_19 - A08 | 1.5 | ant16_21 - D10 | 84 | ant552_360 - C09 | UN | ant567_733 -E06 | UN |

| **IL3Rra1** | | Sigrr | | Ngfr | | CD22 | |
| --- | --- | --- | --- | --- | --- | --- | --- |
| **Antibody** | **Score** | **Antibody** | **Score** | **Antibody** | **Score** | **Antibody** | **Score** |
| ant568_734 - B08 | 273 | ant575_741 - G05 | 199 | ant54_71 - E06 | 111 | ant582_777-B09 | 304 |
| ant568_734 - F11 | 220 | ant575_741 - C01 | 135 | ant54_71 - C09 | 105 | ant582_777 -C08 | 252 |
| ant568_734 - B04 | 192 | ant575_741 - C09 | 102 | ant54_71 - G02 | 101 | ant582_777 -A08 | 222 |
| ant568_734 - H11 | 131 | ant575_741 - B02 | 76.8 | ant54_71 - C07 | 86.3 | ant582_777 -A03 | 216 |
| ant568_734 - B09 | 117 | ant575_741 - C12 | 55.1 | ant54_71 - H02 | 53.6 | ant582_777 -C12 | 182 |
| ant568_734 - F02 | 101 | ant575_741 - G12 | 44.4 | ant54_71 - D03 | 44.8 | ant582_777 -B06 | 159 |
| ant568_734 - F10 | 91.9 | ant575_741 - A01 | 34.5 | ant54_71 - B07 | 29.6 | ant582_777 -C10 | 72 |
| ant568_734 - B06 | 75.4 | ant575_741 - D07 | 26.1 | ant54_71 - F09 | 18.7 | ant582_777 -E03 | 19 |
| ant568_734 - E01 | 48.5 | ant575_741 - D01 | 24.3 | ant54_71 - B06 | 9.4 | ant582_777 -H08 | 4.8 |
| ant568_734 - E12 | 21.6 | ant575_741 - C04 | 8.4 | ant54_71 - B03 | 3.1 | ant582_777 -B05 | 1.7 |

| **VCAM** | | Jagged-1 | |
| --- | --- | --- | --- |
| **Antibody** | **Score** | **Antibody** | **Score** |
| ant588_783 - D03 | 117 | Jag-A06 | 182 |
| ant588_783 - E11 | 63 | Jag-D05 | 177 |
| ant588_783 - D08 | 59.2 | Jag-D07 | 158 |
| ant588_783 - C09 | 53.6 | Jag-A01 | 153 |
| ant588_783 - D04 | 40.2 | Jag-C07.1 | 109 |
| ant588_783 - E02 | 10.3 | Jag-F08 | 99.7 |
| ant588_783 - C11 | 9.44 | Jag-C05 | 33.6 |
| ant588_783 - B04 | 7.96 | Jag-A01.2 | 22.6 |
| ant588_783 - D01 | 2.07 | Jag-E03 | 21.8 |
| ant588_783 - A01 | UN | Jag-A06 | 182 |
