# Supplementary material for: Application of phage display to high throughput antibody generation and characterization
Source: Genome Biol. 2007 Nov 29;8(11):R254. doi: 10.1186/gb-2007-8-11-r254 (PMC2258204; doi:10.1186/gb-2007-8-11-r254)
Supplement: Additional data file 4 — Construction and rescue of the 'McCafferty' antibody phage display library [file gb-2007-8-11-r254-S4.doc]

**Additional data file 4**

**Supplementary Table 3. Description of staining profiles supported by multiple antibodies in immunohistochemistry**

| **antigen** | **clone name** | **Description** | **Supporting data** |
| --- | --- | --- | --- |
| Bsg | ant43_59_C10 | Testis, seminiferous tubules, moderate staining of spermatid tails observed | Present in murine spermatogenesis (Maekawa M, Suzuki-Toyota F, Toyama Y et al. Arch Histol Cytol, 1998, 61: 405). The staining patterns match those described by Nakai M, Chen L, Nowak RA. Annat Record, 2006, 288: 527 |
|  | ant43_59_C12  ant43_59_D08  ant43_59_E02 | Moderate staining of spermatid tails and of seminiferous tubule capsule in testis; cytoplasmic staining of epithelial cells in collecting ducts of salivary gland, mucosa of oesophagus and stomach, lining several renal tubules in kidney; mod/strong staining of external lamina of muscle fibres in smooth and striated muscle, where present, and heart; sinuses of liver; cytoplasmic staining of acinar cells in salivary gland; stroma in mucosa of stomach; Saggital brain shows moderate staining of meninges and/or blood vessels; E14.5 embryo shows staining in liver and lung |  |
|  |  |  |  |
|  |  |  |  |
|  | ant43_59_G05 | Spermatid tail staining again observed plus wk/mod cytoplasmic staining of epithelial cells in the collecting ducts of salivary gland. |  |
| Cd226 | ant348_228_B03 | Few cells/structures in lymph node are moderately stained. In spleen several cells are strongly stained. In large and small intestine cells present in the lamina propria are strongly stained. Non specific staining is present in kidney and liver. Also lymph node for ant348_228_H06. | CD226 is present on human mast cells, T cells, NK cells, dendritic cells in paracortical areas, platelets and megakaryocytes (Kojima H, Kanada H, Shimizu S, et al. J Biol Chem, 2003, 278: 36748, Pende D, Castriconi R, Romagnani P, et al. Blood, 2006, 107: 2030, Bachelet I, Munitz A, Mankutad, Levi-Schaffer F. J Biol Chem, 2006, 281: 27190) |
|  | ant348_228_B04 |  |  |
|  | ant348_228_H06 |  |  |
| CD5 | ant163_154_C10 | ant165_155_A02 shows the most extensive staining: Lymph node, several lymphoid cells show strong staining of scattered cells, probably lymphoid, in the stroma of several tissues to include spleen, lung, thyroid, prostate, bladder, liver, and lamina propria of sm/lge intestine was also observed. Weak connective tissue and pigment staining present. ant165_155_E04 gave moderate to strong membranous staining of lymphoid cells where present: Lymph node extensive staining observed; Spleen, several scattered cells. Lymphoid infiltrates in lung, bladder, thyroid, oesophagus, uterus, ovary and stomach. Scattered cells in stroma of kidney, bladder, thyroid, oesophagus, uterus, cervix, ovary, fallopian tube, skin, stomach and lge/small intestine and ureter. ant163_154_C10, ant165_155_D07 ant165_155_E02 mainly demonstrate staining in L. node and spleen. ant165_155_E05, ant165_155_F06 gave strong granular staining of occasional cells, some of which appear dendritic, observed in Lymph node, spleen, bladder, prostate and uterus and of alveolar macrophages in lung. Wk/mod connective tissue staining present. Mod/strong luminal staining of renal tubules in kidney | CD5 is expressed on T cells and subsets of mature B cells.  (Leukocyte and stromal cell molecules: The CD markers (2007) ed. Zola, Swart, Nicholson, Voss. Wiley ISBN-13:978-0-471-70132-3) |
|  | ant165_155_A02 |  |  |
|  | ant165_155_D07 |  |  |
|  | ant165_155_E02 |  |  |
|  | ant165_155_E04 |  |  |
|  | ant165_155_E05 |  |  |
|  | ant165_155_F06 |  |  |
| CD83 | ant159_152_A05 | ant159_152_A05 weak to moderate staining of scattered cells in spleen and lamina propria of small and large intestine. ant159_152_C10 staining of scattered cells in lymph node, lamina propria of sm/lge intestine and pancreas. Pigment staining present. ant159_152_F11 extensive staining is present including many epithelial components of tissues. In lymph node, many cells are stained this may be either membrane associated or non-specific. Seminiferous tubules in testis are stained and heart muscle is also stained. Patchy adrenal staining is present and placental trophoblasts are intensely stained. Also lamina propria of sm/lge intestine | Marker for mature dendritic cells that stimulate T and possibly IgD+ B cells. In lymph node they are present in paracortical (T cell) and in small numbers in the B cell mantle and primary follicles. They are not present in germinal centres (Takahashi K, Kenji A, Norihiro T, et al. Am J Pathol, 2001, 159:131). In human tonsil they represent one of 5 dendritic cell subsets and a present in the minority in the paracortical regions (Summers KL, Hock BD, McKenzie JL, Hart DNJ. Am J Pathol, 2001, 159: 285). In murine spleen they are present as several isolated cells around the periarteriolar sheath in the white pulp (Wolenski M, Cramer SO, Ehrlich S, et al. Med Microbiol Immunol, 2003, 192: 189). |
|  | ant159_152_C10 |  |  |
|  | ant159_152_F11 |  |  |
| Elf1 | ant78_118_G04 | Bladder epithelium  choroid of sagittal brain (ant78_118_G04) | No supporting evidence. In contrast, staining of a single anti-human ELF1 antibody (ant46_62_F12) showed the expected widespread nuclear staining in several tissues: This includes kidney, renal tubules; adrenal, groups of cells; thyroid follicular cells; oesophagus, epithelial cells; testis, seminiferous tubules, spermatogenic cells; uterus, uterine glands; fallopian tube, epithelium cells; placenta, trophoblastic cells; skin, epidermis; ureter, epithelium; stomach, large and small intestine, mucosa, epithelium; cerebrum, cerebellum and spinal cord, neural and neuroglial cells. Cytoplasmic staining of alveolar macrophages in lung; several scattered cells in spleen and lymph node; several cells in lamina propria of large and small intestine and acinar cells in pancreas is present. In liver the sinusoids are stained and in prostate stromal staining is present. |
|  | ant78_118_H06 |  |  |
| Epha2 | ant3_7_D07 | Show occasional cells in lymphoid tissues, and a reaction in dermis of skin. Some other epithelial reaction is present though not consistently with these antibodies and cells in the lamina propria of small intestine were stained with ant3_7_D07 and ant3_7_E07. ant3_7_G07 weak epithelial staining of many tissues together with a stromal, possible capillary, reaction in skin and striated muscle. | Expressed most highly in tissues that contain a high proportion of epithelial cells, e.g., skin, intestine, lung, and ovary (OMIN and SOURCE databases Immunohistochemical analysis detected for rat protein in lung and kidney epithelial cells (OMIM)  <http://source.stanford.edu/cgi-bin/source/sourceSearch> http://www.ncbi.nlm.nih.gov/entrez/query.fcgi?db=OMIM |
|  | ant3_7_E07 |  |  |
|  | ant3_7_G05 |  |  |
|  | ant3_7_G07 |  |  |
| Ngfr | ant54_71_C07 | ant54_71_C07 peripheral nerve staining is present in several adult cores. Large ducts in salivary gland, stromal staining in uterus and occasional clustered staining in sagittal brain is also present. E14.5 embryo the edge of CNS and spinal cord, choroid, dorsal root ganglia and large nerve tracks are strongly stained. ant54_71_E06 has same distribution of staining but of weaker intensity than ant54_71_C07. ant54_71_G02 is as ant54_71_C07with additional strong staining of few cells in lymph node, spleen, lamina propria of large and small intestine together with weak to strong staining of basal regions of tracheal epithelium | Numerous cell types including schwann cells motor neurons and cerebellar Purkinje cells.  Bothwell M (1991) Tissue localization of nerve growth factor and nerve growth factor receptors  Current Topics in Microbiology and Immunology 165, 55-70  p75 and Trk: A two-receptor system   Chao MV, Hempstead BL (2005) Trends in Neurosciences **18** 321-326 |
|  | ant54_71_E06 |  |  |
|  | ant54_71_G02 |  |  |
| Rab9B | ant308_180_C09 | ant308_180_C09: Axon staining is present in peripheral nerve bundles in many cores. Extremely intense grey matter in spinal cord with staining also in white matter is present. Intense regional (nerve fibre) staining is present in sagittal brain. In E14.5 embryo there is staining of the periphery of the spinal cord and selective staining of moderate to strong intensity in the CNS.  ant308_180_C12 Extremely intense grey matter in spinal cord with staining also in white matter is present. Intense regional (nerve fibre) staining is present in sagittal brain Much staining is present in E14.5 embryo but this may not be specific. Also strong selective staining of renal tubules, basal lamina of large vessels and bronchi in lung, reticulin like staining in adrenal medulla and cortex, staining of bladder wall smooth muscle, cells at edge of islets of Langerhans, basal lamina of seminiferous tubules and peripheral nerve bundles is present. | RAB9 has been localised to components of the endocytic/exocytic pathway and is implicated in the recycling of membrane receptors. RAB9B was identified by cDNA cloning and shown by Northern blotting to widely expressed (Seki N, Azuma T, Yoshikawa T, et al. J Hum Genet, 2000, 45:318). |
|  | ant308_180_C12 |  |  |
| TNFRSF14 | ant53_70_D08 | Moderate to strong staining of scattered cells in spleen and lamina propria of lge/small intestine observed.  ant53_70_D08, ant53_70_D10, ant53_70_E10, ant53_70_F03 show few cells in lymph node, cells in stroma of bladder and fallopian tube, Occasional clustered cells in adrenal are also stained. | Is important in activation of T cells and is expressed on these, B cells, dendritic cells and activated macrophages (Croft M. Nat Rev Immunol, 2003, 3: 609). By Northern blot it is expressed at highest levels in lung, spleen, thymus, CD4+ and CD8+ T cells, CD19+ B cells and monocytes (Marsters SA, Ayres TM, Skubatch M, et al. J Biol Chem, 1997, 272: 14029. Kwon BS, Tan KB, Ni J, et al. J Biol Chem, 1997, 272: 14272). |
|  | ant53_70_D10 |  |  |
|  | ant53_70_E10 |  |  |
|  | ant53_70_F03 |  |  |
|  | ant53_70_H07 |  |  |
|  | ant53_70_H12 |  |  |
| TNFRSF18 | ant52_69_E05 | Few cells in lymph node, occasional cells in stroma of bladder and spleen, cells in lamina propria of large and small intestine and stomach are strongly stained. | TNFRSF18 is expressed at constitutively high levels in CD4+/CD25+ and CD8+/CD25+ T regulatory cells and is up-regulated upon activation. It is also upregulated on CD4+ and CD8+ cells after activation. It is probably crucial in the control of immune system homeostasis (Nocentini G, Riccardi C. Eu J Immunol, 2005, 35: 1016) |
|  | ant52_69_E10 |  |  |
| Tnfrsf19 | ant118_77_A02 | ant118_77_A02 distinct stromal/connective tissue staining observed in all tissues. Extracellular matrix shows an intertwined membranous-type staining of moderate/strong intensity. Blood vessel walls and capsules, where present, show moderate staining. ant118_77_H03 is similar but not so extensive and with less intense staining. E14.5 shows extensive stromal staining. Follicular cells in ovary and several stromal cells in endometrium of uterus show mod/strong membranous staining.  ant118_77_C03 follicular cells in ovary and several stromal cells in endometrium of uterus show mod/strong membranous staining. In liver intense staining of hepatocytes surrounding central veins in liver is present. Delicate staining of alveolar cells in lung is present. In kidney moderate staining of renal tubules is present. There is possible staining of capillaries in bladder. Leydig cells in testis are moderately stained. Some ducts and glands in salivary gland are moderately stained. In uterus capillaries/cells are stained. There are intense epithelial cells in stomach. Cells in grey matter of spinal cord and many cells in sagittal brain are stained. Capillaries and other staining are present in E14.5 embryo. At 0.5 ug/ml specific staining in Leydig cells and uterus is lost though spinal cord and sagittal brain staining may be clearer | By mRNA dot blots shown to be present in many tissues but not lymphoid (Hu S, Tamada K, Ni J, et al. Genomics, 1999, 62: 103., Kojima T, Morikawa Y, Copeland NG, et al. J Biol Chem, 2000, 275: 20742. IHC and ISH expression in developing and adult brain (precursor cells in RMS and SVZ and astrocytes), embryonic epithelium and hair follicles in adult mouse (Pispa J, Mikkola ML, Munstonen T, Thesleff I. Gene Expression Patterns, 2003, 3: 675, Hisaoka T, Morikawa Y, Senba E. Brain Res, 2006, 1110: 81. |
|  | ant118_77_C03 |  |  |
|  | ant118_77_H03 |  |  |
| TNFRSF25 | ant29_42_A07 | ant29_42_A07 gives kidney, lung, lymph node, bladder, pancreas, spleen, thyroid, and lamina propria of stomach and lge/small intestine show strong cytoplasmic staining of several scattered cells. Cells large and sometimes dendritic in appearance, possible demonstration of macrophages. Marked amorphous staining observed in the lumen of several renal tubules in kidney and acini in pancreas, and of colloid present in thyroid follicles. ant29_42_A10 as with ant29_42_A07 with additional course granular cytoplasmic staining of mod/strong intensity observed in epithelial cells of the mucosa of fallopian tubes and syncytotrophoblastic cells of the placenta. ant29_42_C01 as with ant29_42_A07 but with increased staining intensity. In addition foamy cytoplasmic staining of moderate intensity observed in epithelial cells of the mucosa of lge/small intestine. | Otherwise known as LARD, APO3/ Expressed in spleen, thymus and peripheral blood lymphocytes and lower expression in small intestine, colon, foetal lung and foetal kidney. Possible involvement in regulating lymphocyte homeostasis |
|  | ant29_42_A10 |  |  |
|  | ant29_42_C01 |  |  |
| Tnfrsf5 | ant28_41_A01 | Isolated cells in lymph node, spleen and lamina propria of small intestine | Otherwise known as CD40. Expressed on all mature B cells, pro-B through to plasma cells. Present also on monocytes, DC’s, endothelial and epithelial tissues, basal epithelial cells, endothelial cells, fibroblasts, keratinocytes, interdigitating cells and CD34 haematopoeitic progenitor cells. (Leukocyte and stromal cell molecules: The CD markers (2007) ed. Zola, Swart, Nicholson, Voss. Wiley ISBN-13:978-0-471-70132-3) |
|  | ant28_41_A04 |  |  |
|  | ant28_41_C01 |  |  |
|  | ant28_41_D08 |  |  |
| Tnfrsf6 (Fas) | ant51_68_C11 | Moderate staining of basal region of gastric glands in stomach and in crypts of intestinal glands of small intestine. Pancreas, acinar cells show weak cytoplasmic staining and moderate perinuclear staining. |  |
|  | ant51_68_C12 |  |  |
| Tnfrsf7 | ant24_39_B04 | ant24_39_B04 weak/moderate general staining present with more intense staining of the following: widespread staining of blood vessels and capillaries in several tissues; membranous staining of cells in alveolar walls, possibly endothelial; neural cells in spinal cord and sagittal brain and of nerve ganglia and bundles in stomach, small and large intestine; punctate-like staining in seminiferous tubules of testis, possibly represent spermatid heads; cytoplasmic staining of cells present in dermis of skin, spleen trabeculae and connective tissue of oesophagus, uterus and ovary. HIGH signal/noise suggests repeat at lower antibody concentration. ant24_39_E05 gives widespread staining of blood vessels and capillaries observed in thymic medulla, stomach, oesophagus, skin and pancreas. Scattered cells in the lung, possibly alveolar macrophages, Cells present in white pulp of spleen and show mod/strong cytoplasmic staining. Peripheral nerves, where present, show mod staining of perinureum (nerve capsule). Some further staining but looks non-specific. | Otherwise known as CD27. Found on haematoetic stem cells and early progenitor cells, medullary thymocytes, activated B cells and NK cells. Constitutively expressed on T cells with expression elevated on activated CD45RA T cells.  (Leukocyte and stromal cell molecules: The CD markers (2007) ed. Zola, Swart, Nicholson, Voss. Wiley ISBN-13:978-0-471-70132-3) |
|  | ant24_39_E05 |  |  |
| Tnfrsf9 | ant23_36_B04 | All gave patchy, weakk connective tissue staining present in dermis of skin. Moderate cytoplasmic staining of very occasional cells in the medulla of thymus. ant23_36_C02 also gave weak cytoplasmic staining of cells in base of glands in stomach. ant23_36_B04 also gave weak cytoplasmic staining of cells at base of glands in stomach and wk, granular cytoplasmic staining of acinar cells in pancreas. | Otherwise known as CD137. Present on activated T and B cells, monocytes, follicular dendritic cells. |
|  | ant23_36_C02 |  |  |
|  | ant23_36_D05 |  |  |
|  | ant23_36_F06 |  |  |
|  | ant23_36_G11 |  |  |
